# Evolution of *Mycobacterium abscessus* in the human lung: Cumulative mutations and genomic rearrangement of porin genes in patient isolates

**DOI:** 10.1080/21505594.2023.2215602

**Published:** 2023-06-04

**Authors:** Shamira J. Shallom, Hervé Tettelin, Prabha Chandrasekaran, In Kwon Park, Sonia Agrawal, Kriti Arora, Lisa Sadzewicz, Aaron M. Milstone, Moira L. Aitken, Barbara A. Brown-Elliott, Richard J. Wallace, Elizabeth P. Sampaio, Michael Niederweis, Kenneth N. Olivier, Steven M. Holland, Adrian M. Zelazny

**Affiliations:** a Microbiology Service, Department of Laboratory Medicine (DLM), Clinical Center, NIH, Bethesda, MD, USA; b Institute for Genome Sciences, Department of Microbiology and Immunology, University of Maryland School of Medicine, Baltimore, MD, USA; c Laboratory of Clinical Infectious Diseases (LCID), National Institute of Allergy and Infectious Diseases (NIAID), NIH, Bethesda, MD, USA; d Pediatric Infectious Diseases, Johns Hopkins University, Baltimore, MD, USA; e Division of Pulmonary and Critical Care Medicine, University of Washington Medical Center, Seattle, WA, USA; f Mycobacteria/Nocardia Laboratory, University of Texas Health Science Center, Tyler, TX, USA; g Department of Microbiology, University of Alabama, Birmingham, England; h Laboratory of Chronic Airway Infection, Pulmonary Branch, National Heart Lung and Blood Institute (NHLBI), NIH, Bethesda, MD, USA

**Keywords:** Mycobacterium massiliense, Mycobacterium abscessus, cystic fibrosis, Porin, whole genome sequencing, immune response

## Abstract

**Background:**

*Mycobacterium abscessus* subspecies *massiliense* (*M.* *massiliense*) is increasingly recognized as an emerging bacterial pathogen, particularly in cystic fibrosis (CF) patients and CF centres’ respiratory outbreaks. We characterized genomic and phenotypic changes in 15 serial isolates from two CF patients (1S and 2B) with chronic pulmonary M. massiliense infection leading to death, as well as four isolates from a CF centre outbreak in which patient 2B was the index case.

**Results:**

Comparative genomic analysis revealed the mutations affecting growth rate, metabolism, transport, lipids (loss of glycopeptidolipids), antibiotic susceptibility (macrolides and aminoglycosides resistance), and virulence factors. Mutations in 23S rRNA, *mmpL*4, porin locus and *tet*R genes occurred in isolates from both CF patients. Interestingly, we identified two different spontaneous mutation events at the mycobacterial porin locus: a fusion of two tandem porin paralogs in patient 1S and a partial deletion of the first porin paralog in patient 2B. These genomic changes correlated with reduced porin protein expression, diminished ^14^C-glucose uptake, slower bacterial growth rates, and enhanced TNF-α induction in mycobacteria-infected THP-1 human cells. Porin gene complementation of porin mutants partly restored ^14^C-glucose uptake, growth rate and TNF-α levels to those of intact porin strains.

**Conclusions:**

We hypothesize that specific mutations accumulated and maintained over time in *M.* *massiliense*, including mutations shared among transmissible strains, collectively lead to more virulent, host adapted lineages in CF patients and other susceptible hosts.

## 1. Introduction

Nontuberculous mycobacteria (NTM) are ubiquitous environmental organisms that can cause chronic pulmonary infection, particularly in individuals with pre-existing lung disease such as chronic obstructive pulmonary disease (COPD), bronchiectasis, and cystic fibrosis (CF) [1,2]. Among NTM, *M. abscessus sensu lato* (*MAB*) is responsible for most lung infections due to rapidly growing mycobacteria (RGM) and includes the *M. abscessus* subspecies *abscessus* (referred to here as *M. abscessus*), *M. abscessus* subspecies *bolletii* (*M. bolletii*), and *M. abscessus* subspecies *massiliense* (*M. massiliense*). *MAB*, a leading cause of NTM lung infection, is clinically resistant to most antibiotics and difficult to cure [1,3]. It is also a major pulmonary pathogen in patients with CF, where it is associated with significant morbidity and mortality [3–5]. *M. massiliense* has been also reported to cause outbreaks of soft tissue lesions [6–8] and pulmonary disease among patients attending two CF centres in the United States (Seattle) and the United Kingdom [9,10] with evidence of transmission between patients [6,9,11].

Whole genome sequences (WGS) of NTM isolates are available, providing more information on bacterial genomic diversity and identification of potential virulence factors. Porins are channel-forming proteins that mediate the uptake of small, hydrophilic molecules across bacterial outer membranes, and have been shown to impact nutrient uptake, antibiotic resistance and intracellular survival [12–16]. Following sequencing of mycobacterial isolates, we pursued genomic and phenotypic changes in 11 serially collected clinical isolates from two patients (1S and 2B) with CF who presented with chronic *M. massiliense* pulmonary infection. We observed within-host parallel evolution in patients 1S and 2B with non-synonymous SNPs or indels in both patients in 23S rRNA, *mmpL*4, porin *and tetR* genes. In addition, we characterized porin genes changes genetically and functionally. Our data indicates that *M. massiliense* strains acquire mutations over time within the host leading to antimicrobial resistance, increased pathogenicity, and adaptation.

## 2. Methods

### 2.1. Subjects

Two patients with CF who presented with pulmonary *M. massiliense* infection were followed and treated at the Johns Hopkins University (patient 1S), and at the National Institutes of Health (NIH) and University of Washington (patient 2B). Patient 1S anonymized isolates and clinical data were obtained from another institution post-mortem. Patient 2B was part of a NIH IRB approved protocol. Patient 2B was the index patient in the reported *M. massiliense* respiratory outbreak in a CF centre in Seattle [9]. We obtained serial isolates (Suppl. Table S1) and reviewed medical records from the two CF patients. In addition, four isolates from patients involved in the Seattle CF centre outbreak [6] were also sequenced (Suppl. Table S1). Both patients 1S and 2B had rapid clinical decline leading to death after several years of relatively stable disease course. Two of the other four patients from the Seattle outbreak subsequently died with recalcitrant mycobacterial disease.

**Patient 1S** was a 19-year-old male diagnosed with CF (c.394delTT, c.2184delA). The patient had his first positive mycobacterial culture in 2003, at age 14, identified by bronchoalveolar lavage (BAL) for an episode of pneumonia. Patient 1S was infected with methicillin susceptible *Staphylococcus aureus* and *Pseudomonas aeruginosa* prior to isolation of his first mycobacterial strain 1S–1. Subsequent respiratory cultures grew *M. massiliense*, although he was doing well clinically. From 2006, his clinical status began to decline and within one year, his FEV_1_ decreased. He was hospitalized in 2007 and BAL culture grew *M. massiliense* as well as *Stenotrophomonas maltophilia*. He was discharged home on azithromycin, amikacin, and imipenem. With stabilization in his status, the regimen was simplified to azithromycin and inhaled amikacin. One year later, he had a rapid decline, requiring intensive care unit admission and subsequently died of an overwhelming mycobacterial pulmonary infection.

**Patient 2B** was a 22-year-old male with CF (p.508delF, c.1717-1 G>A) who had his first *M. massiliense* isolated from BAL in 2000, at age 15. Patient 2B was infected with *Pseudomonas aeruginosa* and *Aspergillus fumigatus* in childhood, long before strain 2B–1 was isolated. He did well on ertapenem, linezolid, moxifloxacin, and azithromycin for several years, maintaining an active lifestyle. In early 2007 his clinical status started to decline; he received a double lung transplant in late 2007 but died of disseminated *M. massiliense* infection at day 74 post-transplant in 2008. He was the index patient in the reported *M. massiliense* respiratory outbreak in a CF centre in Seattle, USA involving a total of five patients with three deaths [6].

### 2.2. Bacterial isolates: culture, growth conditions and antibiotic susceptibility testing

Mycobacterial clinical isolates collected serially from the patients throughout their disease courses, from chronic stable to terminal stages, were available for whole genome sequencing (WGS). For patient 1S, a total of four isolates were collected over a five-year period and available for WGS. For patient 2B, 11 isolates collected over an eight-year period and seven of them were whole genome sequenced. In addition, four isolates from patients involved in the Seattle CF centre outbreak [6] were also sequenced (Suppl. Table S1).

Mycobacteria were grown in Middlebrook 7H11 agar (Remel, Lenexa, KS) for 3–5 days at 35–37°C, CO_2_ incubator. For experiments requiring liquid cultures, the isolates were grown on Middlebrook 7H9 broth supplemented with ADC (albumin, dextrose, and catalase) enrichment broth (BD, Franklin Lakes, NJ). Tween 80 (0.05%) and glycerol (0.2%) were added to the broth to minimize clumping. For long-term storage, bacterial strains were kept at −70°C in Tween albumin broth (Remel, Lenexa, KS). All isolates were identified at subspecies level, *M.* *abscessus* subspecies *massiliense*, as described previously [17] (data not shown).

For mycobacterial growth curve experiments, serial clinical isolates were inoculated into Middlebrook 7H9 liquid medium (with 0.05% Tween 80, 0.2% glycerol and ADC-10% vol was added after the media was sterilized), and the cultures allowed to grow at 35–37°C (non-CO_2_ shaker) for 2–3 days. This starter culture was used to inoculate 50 ml broth (initial OD_600_ of 0.025) and OD_600_ recorded every 12 h. Growth curves of all serial isolates from patient 1S and 2B were performed in two biological replicates with technical replicates. Growth curves for key isolates: 1S–1, 1S–4, 2B–1, 2B–5, and 2B–11 were repeated with additional biological replicates along with porin plasmid complemented and vector complemented isolates (data not shown). Statistical analysis was done using a two-way ANOVA with Bonferroni multiple comparisons test, to determine statistical significance at *p*-value <0.05 for time related growth rate measurements. Antibiotic susceptibility was done using RAPMYCO plates with CAMHB (Cation Adjusted Mueller Hinton Broth) in duplicates and incubated at 30°C non-CO_2_ incubator (ThermoFisher Scientific, Asheville, NC) according to CLSI 1^st^ Edition M62 for MIC interpretations, QC (quality control) etc [18].

### 2.3. DNA isolation, PCR, mycobacterial whole genome sequencing and annotation

DNA for whole genome sequencing was extracted using the CTAB-NaCl method [19]. The draft genomes of the 10 clinical *M. massiliense* isolates (1S–1, 1S–2, 1S–3, 1S–4, 2B–1, 2B–4, 2B–6, 2B–7, 2B–9, and 2B–11) were sequenced using a combination of 454 paired-end technology and Illumina HiSeq 2000 technologies to achieve high genome sequence quality given their large genome sizes and high G+C content. The 454 paired-end technology was used to generate ~ 20× coverage of each genome from 3Kb insert libraries, and the Illumina HiSeq 2000 100bp paired-end technology was used to generate > 100× coverage of each genome from 340bp insert libraries. Isolate 2B–5 was sequenced later using Illumina Hiseq4000 150bp paired end technology. The Illumina read coverage was sub-sampled during assembly (Celera Assembler v6.1 or v7.0) to 20-60× to achieve optimal assembly results for each genome. The four isolates from the Seattle outbreak were sequenced with the single molecule real-time (SMRT) Pacific Biosciences sequencing technology to a coverage of 10-25× and assembled with HGap assembler v1.4 or Celera Assembler v7.0. Sets of contigs obtained for each genome were annotated using the Institute for Genome Sciences (IGS) Annotation Engine automated annotation pipeline [20]. Mycobacterial serial patient isolates and 4 Seattle outbreak isolates are listed in (Suppl. Table S1) along with strain designations and accession numbers for genomes deposited in GenBank and the corresponding label of each isolate used throughout this study.

For PCR and targeted sequencing of the clinical isolates, DNA was extracted with the Ultra Clean microbial DNA isolation kit, according to the manufacturer’s instructions (Mol Bio Laboratories, Solana Beach, CA). Genomic changes in the porin locus (paralogs) were assessed by PCR using PrimeSTAR Max DNA Polymerase (Takara, San Jose CA). The primers (A-short-F and Porin 2B) listed in Suppl. Table S3 were used to amplify the porin region in patient 1S isolates and sequence the region. To capture the deletion region in the first copy of the porin gene and the upstream region in patient 2B, the following two primers 2B_0626_UP4F and Porin_int780_RC (primer in the intergenic region between the two porin paralogs) were used to generate the PCR product and for sanger sequencing. Primers for 23S rRNA and 16S rRNA [21] were used to determine mutations by sanger sequencing (Suppl. Table S3).

### 2.4. Core genome phylogenetic analysis, prediction of insertions, deletions, and frameshift mutations

The annotated contigs from our draft genomes were combined with other publicly available complete and draft whole genome sequences from the *M. abscessus* group including the *M. abscessus* and *M. bolletii* type strains. All selected genome sequences were subjected to whole genome multiple sequence alignments using the Mugsy software [22] with default parameters. The alignment in MAF format was then filtered with Phylomark [23] to extract and concatenate the core nucleotides including SNPs and to construct a neighbour-joining phylogenetic tree using MEGA v5.1 [24]. The alignment in MAF format was also used in conjunction with annotation for each genome to generate Mugsy clusters of syntenic orthologs [25]. These Mugsy clusters were used for identification of genes whose presence/absence pattern or structure (truncation, point mutation, frame shift) varied between isolates. Comparative genomic data were interrogated using the Sybil platform [26].

### 2.5. Cloning of porin paralogs and complementation of the gene in porin mutant mycobacterial isolates

**Patient 1S, porin complementation in isolate 1S–4**: The porin locus comprising the two copies of the porin gene, and the intergenic region was PCR amplified using PrimeSTAR Max DNA Polymerase (Takara, San Jose CA), using isolate 1S–1 as a template (94ºC for 3 min, followed by 35 cycles of 94ºC for 30 secs, 67ºC for 30 secs and 72ºC for 2 min, followed by 72ºC, 10 min) with primers PorinF_HindIII and Porin2R_HindIII (Suppl. Table S3). The hygromycin resistance gene in the red fluorescent protein plasmid pCherry3 (#24659, Addgene, Watertown MA) [27] was replaced by the kanamycin resistance selection marker (a kind gift from Dr. M. Jackson, University of Colorado, Fort Collins, CO). The pCherry3-Kan plasmid was digested at the BamHI (NEB, Ipswich, MA) restriction site, the ends were blunt-ended with T4 DNA polymerase (NEB, Ipswich MA) and treated with CIP (Alkaline phosphatase Calf Intestinal, NEB, Ipswich MA) to prevent re-ligation of the vector. The porin locus (PCR amplified from 1S–1) was blunt end ligated using Blunt/TA ligase (NEB, Ipswich MA) into the pCherry3-Kan plasmid at the blunt-ended BamHI restriction site. The construct was transformed into chemical competent DH10B cells (Invitrogen, Thermo Fisher Scientific, Waltham, MA), colonies were picked, and the plasmid was purified using miniprep kit from Qiagen. The orientation of the porin locus insert was determined by restriction digest with XbaI and BamHI and by sequencing of the clone. Large scale plasmid preparations were made using a plasmid preparation kit (Qiagen, Germantown, MD). The last isolate from patient 1S with the porin mutation (1S–4) was made competent and complemented by transformation with the pCherry3-Kan plasmid with the cloned porin locus as described [28].

**Patient 2B, porin complementation in isolate 2B–5 and 2B–11**: The pCherry3-Hyg plasmid [27] with the hygromycin resistance cassette (Plasmid # 24659, Addgene, Watertown, MA) [27] was restriction digested with BamHI and HindIII (NEB, Ipswich MA), the ends were blunt-ended with T4 DNA polymerase (NEB, Ipswich MA) and treated with CIP (Alkaline phosphatase Calf Intestinal, NEB, Ipswich MA) to prevent re-ligation of the vector. The porin locus (PCR amplified from 2B–1) was blunt end ligated using Blunt/TA ligase (NEB, Ipswich MA) into the pCherry3-Hyg plasmid at the blunt-ended BamHI-HindIII restriction site. The construct was transformed into chemical competent DH10B cells (Invitrogen, Thermo Fisher Scientific, Waltham, MA). Plasmid was purified using a miniprep kit (Qiagen). The orientation of the insert was determined by restriction digest with XbaI and BamHI and by sequencing of the clone. pCherry3-Hyg plasmid with the cloned porin locus was transformed into competent isolates 2B–5 and 2B–11 as described [28]. Plasmid pCherry3-Hyg with the hygromycin resistance cassette was used for isolates 2B–5 and 2B–11 since the isolates were resistant to kanamycin.

### 2.6. Glucose uptake assay

Glucose uptake measurements were done on naive isolates (1S–1, 1S–4, 2B–1, 2B–5 and 2B–11) as well as isolates complemented with the porin plasmid or vector alone (1S–4 porin, 1S–4 vector, 2B–5 porin, 2B–5 vector, 2B–11 porin and 2B–11-vector) by growth in Middlebrook media to an OD_600_ of 0.7 in the presence of 1 mM glucose as previously described [29]. In short, bacteria were harvested by centrifugation at 5,000 rpm for 15 min at 4**°**C, washed twice in the uptake buffer (2 mM PIPES, pH 6.5, 0.05 mM MgCl_2_), and incubated at 37**°**C in the buffer containing 20 μM of ^14^C-glucose (Perkin Elmer, MA) for upto 25 minutes. ^14^C-glucose uptake was stopped using the kill buffer (0.1 M LiCl, 6.7% formalin) and bacteria were washed twice to remove the unbound glucose. Cells were counted on a liquid scintillation counter. Experiments were performed in cultures from three biological replicates with two technical replicates. Dry weight of the pellets was assessed for each tube and uptake levels were normalized and expressed as nM/mg of bacteria. Statistical analysis was done using a two-way ANOVA with Bonferroni multiple comparisons test to determine statistical significance at p-value <0.05.

### 2.7. Expression of the porin protein

To evaluate the expression of porin, bacterial protein lysates were extracted as previously described [30]. Briefly, mycobacteria grown in 7H9 broth with ADC were spun (3000 rpm for 15 minutes), washed twice, and their wet weight measured. Bacteria (10 mg) were suspended in 150 μl PG05 buffer (100 mM Na_2_HPO_4_/NaH_2_PO_4_, 150 mM NaCl, 0.1 mM EDTA, 0.5% Genapol, pH 6.5), and incubated for 30 min at 100°C. The lysates were cooled on ice, spun at 10,000 rpm (10 min, 4°C) to remove debris, and protein concentration assayed using the BCA method following the manufacturer’s protocol (BioRad Laboratories, CA). Equal amounts of protein were added to each well, resolved on SDS-PAGE, and immunoblotted utilizing a rabbit polyclonal antibody against Porin [31].

### 2.8. In vitro infection of human macrophages and immunological assay.

Patient 1S’s first and last isolates 1S-1and 1S–4, and 1S–4 porin complemented and 1S–4 vector isolates as well as patient 2B first and last isolates 2B–1 and 2B–11, and 2B–11 porin and 2B–11 vector isolates, were grown in a small starter culture. Serial clinical isolates were inoculated into liquid medium (Middlebrook 7H9 with 0.05% Tween 80, 0.2% glycerol and ADC), and the cultures allowed to grow at 35–37 °C for 2–3 days. This starter culture was used to inoculate 50 ml broth THP-1 cells cultured in RPMI media (ThermoFisher Scientific, Asheville, NC) supplemented with 10% heat inactivated FBS and a combined 1× concentration of Penicillin (100 Units/ml), Streptomycin (100 μg/ml), L-Glutamine supplement (0.29 mg/ml) (ThermoFisher Scientific, Asheville, NC). To assess cytokine production, THP-1 cells (ATCC TIB-202) 0.25 × 10^6^/ml in RPMI 1640 medium with GlutaMAX supplement (ThermoFisher Scientific, Asheville, NC) with 10% FBS were stimulated with phorbol-12-myristate-13-acetate (PMA) at the concentration of 10–15 ng/ml for 18–24 hours to allow the cells to adhere on a 24 well plate. Non-adherent cells and PMA were removed with two washes with RPMI 1640 medium with GlutaMAX supplemented with 10% FBS, and the monolayer of cells was rested for 48 hours until they differentiated into macrophage-phenotype. Three hours before infection the macrophages were washed with RPMI 1640 Glutamax medium (without addition of antibiotics) supplemented with 10% FBS and fresh medium was added. These macrophages were infected with first and last *M. massiliense* isolates from patients 1S (1S–1, 1S–4) and 2B (2B–1, 2B–11) and isolates complemented with the porin plasmid or empty vector (1S–4 porin, 1S–4 vector, 2B–11 porin and 2B–11 vector) at a MOI (multiplicity of infection) of 1:5 for 24 hours. The supernatants for the infected cells were collected at 24 hours and stored at −80°C until assayed.

Cytokine levels were measured in supernatants by a Bioplex bead-based assay comprising TNF-α (BioRad, CA). The cytokine assay was processed according to the manufacturer’s specifications. The data represent the mean±SEM of five separate biological replicates with two technical replicates for each isolate. Statistical analysis was done using one-way ANOVA and paired t-tests using the last isolates from patient 1S (1S–4) and patient 2B (2B–11) as anchors with multiple comparisons test performed by the FDR method (Benjamini, Krieger and Yekutieli) with FDR set at 0.05.

## 3. Results

### 3.1. Characteristics of patient lung function and drug treatment

Serial measurements of forced expiratory volume in one second (FEV_1_) were recorded as part of evaluation of lung function. For patient 1S (Suppl. Figure S1A) baseline FEV_1_ measured at the first encounter 1 month after the first positive isolate in BAL was 3.48 litres (isolate 1S–1 collected). The patient remained relatively stable for ~3.0 years (post first positive culture), with FEV_1_ of 3.74 L at 0.7 years, 3.38 L at 1.2 years and 3.36 L at 3.2 years. After 3 years, the patient’s health declined requiring an ICU admission. His FEV_1_ decreased rapidly from 3.36 L (at 3.2 years) to 1.87 L around the 4.2-year mark which recorded at the time isolate 1S–3 was collected and he was hospitalized. The patient was discharged home on azithromycin, and intravenous amikacin and imipenem (unfortunately, we do not have record of antibiotic treatment earlier in the patient course). With stabilization in his status, the regimen was simplified to azithromycin and inhaled amikacin. The final FEV_1_ recorded for patient 1S was 1.58 L, recorded at the 5.0-year mark and a month later the last isolate 1S–4 was collected (5.2 years), shortly before death of patient 1S of an overwhelming mycobacterial pulmonary infection.

Patient 2B did well on the regimen including ertapenem, linezolid, moxifloxacin, and azithromycin for several years (Suppl. Figure S1B). Patient 2B (post first positive culture) had a recorded FEV_1_ value of 2.24 L, 1.5 years after the first positive mycobacterial isolate from BAL specimen was collected (2B–1). The patient remained stable over the subsequent year with ~FEV_1_ of 2.3 L at 2.5 years (isolate 2B–4 was collected) and subsequently moxifloxacin was given. This was followed by a steady decline in FEV_1_ beginning about 5.25 years from his initial isolate to an FEV_1_ of 1.95 L which coincided with a transition from smooth (2B–6) to rough colony morphology (2B–7). We have recorded that the patient received tigecycline, tobramycin and inhaled amikacin in the period between isolates 2B–6/2B–7 and 2B–8 isolation on the timeline. Patient 2B experienced a more rapid decline in lung function in the last year of his life around the collection of 2B–8 isolate. A month later, an FEV_1_ of 1.4 L was recorded when 2B–9 was isolated. The patient received a lung transplantation 10 months after isolate 2B–8 was collected; however, the mycobacterial infection continued, presumably due to reinfection of the graft. The final FEV_1_ recorded for patient 2B was 1.08 L when isolate 2B–11 was collected followed by subsequent death of the patient from disseminated *M. massiliense* infection at day 74 post-transplant (Suppl. Figure S1B).

### 3.2. Serial mycobacterial isolates from patients 1S and 2B: Phenotype, growth rate and antibiotic susceptibility

**Figure 1.**
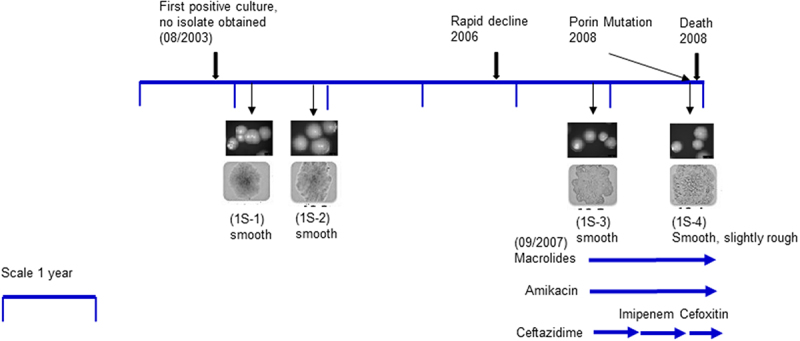
Isolates and timeline of patient 1S: Colony morphology pictures of four patient isolates showed in chronological order: 1S–1, 1S–2, 1S–3, and 1S–4. Upper panels are colony pictures at 10× magnification and lower panels show single colonies at 100× magnification. Isolates 1S–1, 1S–2 and 1S–3 have a smooth morphology, while isolate 1S–4 is smooth albeit slightly rough. The timeline for isolate collection for patient 1S was approximately 5 years. Antibiotic therapy was initiated at a later stage of the disease course around the time of isolate 1S–3.

We gathered clinical mycobacterial isolates and reviewed medical records from the two CF patients with pulmonary *M. massiliense* infection. Both patients had rapid clinical declines leading to death after several years of relatively stable disease courses. Evaluation of mycobacterial growth on solid media revealed subtle changes in colony morphology for patient 1S. Isolates 1S–1 to 1S–3 showed a smooth morphology but the last isolate, 1S–4 was smooth, but had a slightly rougher (grainier) morphology (Figure 1). We also assessed the growth rate of the isolates in broth cultures. Serial isolates from patient 1S (1S–1, 1S–2, and 1S–3) had similar growth rates; the last isolate of patient 1S (1S–4) was slower in growth compared to isolates 1S–1, 1S–2 and 1S–3 and was statistically significant (p-value <0.0001) by 2-way ANOVA Bonferroni multiple comparisons test from timepoint 36 to 72 hours. (Suppl. Figure S2A).

**Figure 2.**
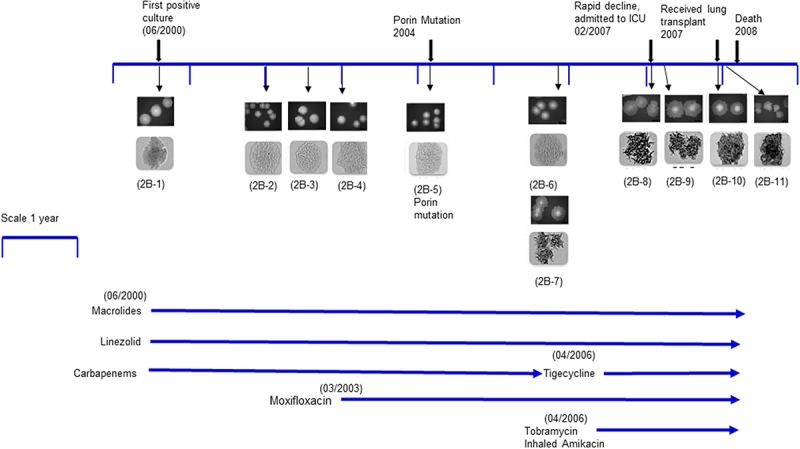
Isolates and timeline of patient 2B: Colony morphology pictures of eleven patient isolates showed in chronological order 2B–1, 2B–2, 2B–3, 2B–4, 2B–5, 2B–6, 2B–7, 2B–8, 2B–9, 2B–10, 2B–11. The first six isolates were smooth. Strain 2B–6 (smooth) and 2B–7 (rough) were coisolated in the same culture. Isolates 2B–7 to 2B–11 were rough. Upper panels are colony pictures at 10× magnification; lower panels show single colonies at 100× magnification. The timeline for isolate collection for patient 2B was approximately 8 years. Antibiotic therapy was initiated when the first isolate 2B–1 was isolated.

Evaluation of colony morphology of patient 2B isolates showed that earlier isolates were smooth (2B–1 to 2B–6) whereas isolate 2B–7 (co-isolated in the same culture along 2B–6) was rough, and the rough morphology continued through the last isolate 2B–11 (Figure 2). However, isolate 2B–11 was notably much rougher and cord-like compared to 2B–7. Patient 2B was clinically stable until co-isolation of 2B–6 (smooth) and 2B–7 (rough) (Figure 2). On comparing isolates from patient 2B, there was a progressive reduction in growth compared to the first isolate 2B–1, from smooth (2B–2, 2B–3, 2B–4, 2B–5, and 2B–6) to the rough isolates (2B–7, 2B–8, 2B–9, 2B–10, and 2B–11). Growth rate experiments in liquid media showed higher growth rates of the smooth isolates (2B–1, 2B–2, 2B–3, 2B–4, 2B–5, and 2B–6) compared to the rough isolates (2B–7, 2B–8, 2B–9, 2B–10, and 2B–11) with progressively diminished growth rate through 2B–11 (Suppl. Figure S2B). These results were confirmed by assessment of colony forming units (data not shown).

### 3.3. Serial mycobacteria isolates obtained from patients 1S and 2B: antibiotic susceptibility

Antimicrobial susceptibility testing of the first isolate 1S–1 from patient 1S (Suppl. Table S2A) showed susceptibility to clarithromycin with a MIC of 0.5 μg/ml at 14 days incubation. In contrast, the last isolate 1S–4 was resistant to clarithromycin at day 3, with a MIC of 16 μg/ml, consistent with acquired resistance. Isolate 1S–1 was susceptible to imipenem (4 μg/ml) and linezolid (8 μg/ml) while the last isolate 1S–4 had intermediate resistance with a 4-fold increase to imipenem (16 μg/ml) and a 2-fold increase to linezolid (16 μg/ml). Amikacin treatment was started around the collection of isolate 1S–3, but the final isolate collected 1S–4 remained susceptible to amikacin with a MIC of 4 μg/ml. Porin gene complementation of isolate 1S–4 showed a mild 2-fold reduction in MIC for imipenem (MIC of 8 versus 16).

Patient 2B first isolate 2B–1 (Suppl. Table S2B) was susceptible to clarithromycin with an MIC of 1 μg/ml at 14 days. Strain 2B–5 (first Porin mutant) and 2B–11 (last isolate) were resistant to clarithromycin at 3 days with an MIC of 16 μg/ml, consistent with acquired resistance [32,33]. Isolate 2B–1 was resistant to imipenem (>64 μg/ml) and linezolid (>32 μg/ml) features which continued through the last isolate 2B–11. Interestingly, phenotypic resistance to amikacin was already present in the first isolate 2B–1 with MIC of >64 μg/ml which remained through the last isolate 2B–11 (Suppl. Table S2B). Porin gene complementation of isolate 2B–5 showed an 8-fold reduction in tigecycline MIC (MIC of 0.25 versus 2) similar to that of 2B–1. However, no change in MIC values were noted upon porin complementation of 2B–11.

### 3.4. Mycobacterial genomes and phylogenetic analysis across serial isolates

**Figure 3.**
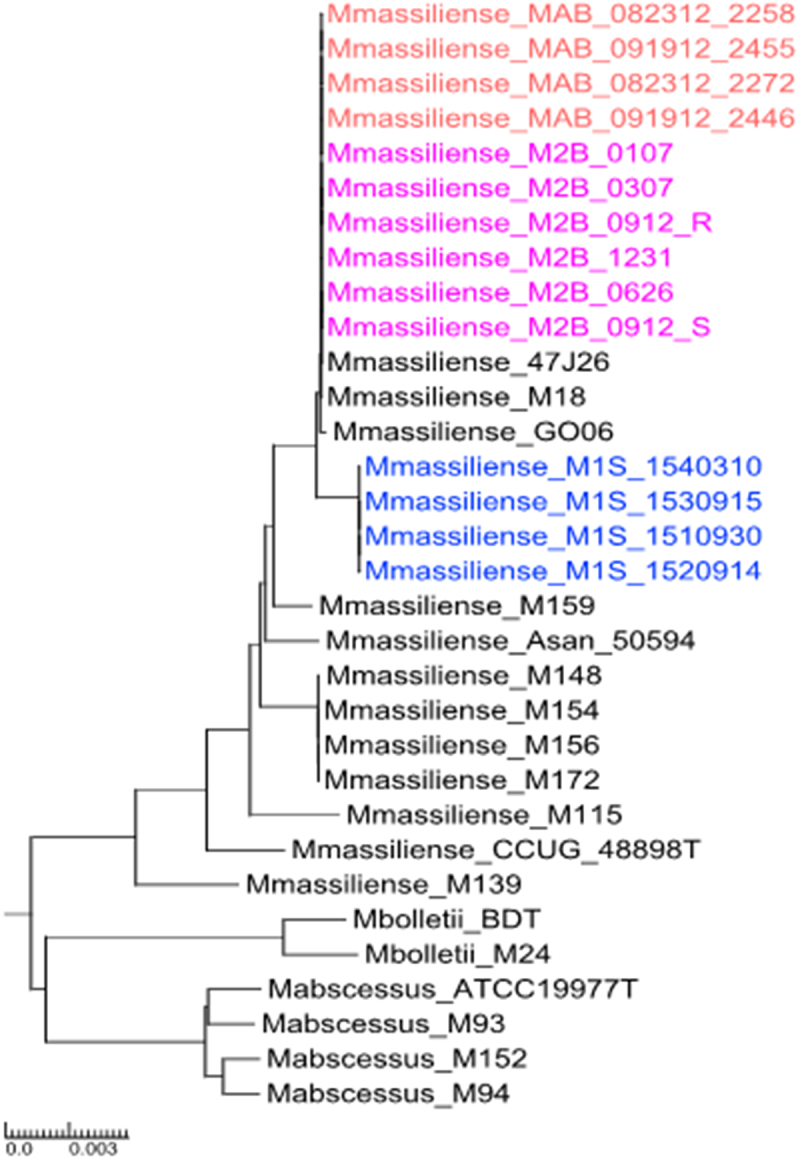
Neighbor-joining phylogenetic tree: Phylogenetic tree based on the whole genome multiple alignment of M. abscessus genomes. The mycobacterial genomes were aligned using Mugsy and core segments were identified using Phylomark. Resulting concatenated nucleotide sequences were used for construction of the mid-point rooted tree using MEGA. Patient 1S isolates are coloured blue, patient 2B isolates are magenta. The outbreak isolates from patient 2B are in orange. Reference genomes are shown in black.

A total of nine isolates from patient 1S were collected over a five-year period and four of the isolates were available at NIH and were whole genome sequenced. A total of 11 isolates collected from patient 2B over an eight-year period were studied and seven of them were subjected to whole genome sequencing (Suppl. Table S1). Genome sequences of the serial *M. massiliense* isolates were assembled into 2 to 13 large contigs. The genomes obtained from patients 1S and 2B consist of circular chromosomes of 4.8–4.9 Mbp including ~ 4,800 predicted open reading frames (ORFs) with a coding capacity of ~ 92% and a GC content of ~ 64%. To investigate further the relationship between our patient’s serial clinical isolates, and 18 publicly available representative genomes of NTM isolates from the *M. abscessus* group, including the type strains for each *MAB* subspecies (*M. abscessus, M. massiliense* and *M. bolletii*), core segments of a whole genome multiple nucleotide alignment were used to construct a neighbour-joining phylogenetic tree (Figure 3). The tree reveals deep branches delineating three major clades that correspond to the three *MAB* subspecies. All isolates from a given patient cluster very closely with each other when compared to other isolates. Average nucleotide identity, which is the similarity index between a given pair of genomes was determined: 1S–1 was 99.9% identical to the last isolate 1S–4; while it was 96.2 % like 2B–1, the first isolate from patient 2B. Similarly, when 2B–1 was used as an anchor, it was 99.9 % identical to 2B–5 and 2B–11.

Isolates from patient 2B (in pink) and from the Seattle outbreak (in orange) appear to be highly related to isolates Birmingham 47J26 (a respiratory isolate obtained from a CF patient [34]) and M18 (isolated from a lymph node biopsy obtained from a patient with cervical lymphadenitis [35], as well as related to soft tissue infection Brazilian outbreak *M. massiliense* isolate GO 06 [36]. Patient 2B was the index case in Seattle CF centre outbreak, which led to spread of isolate 2B–11 to the other four patients. Three isolates from other patients in the outbreak (MAB_091912_2446, MAB_091912_2455, MAB_082312_2258 and MAB_082312_ 2272) [37] cluster on the phylogenetic tree along with the serial isolates from patient 2B.

### 3.5. Key single nucleotide polymorphisms (SNPs) and Indels identified in patients 1S and 2B isolates

High quality non-synonymous SNPs and indels (insertions/deletions) were identified in 15 genes in isolates from patient 1S (8 SNPs and 7 indels), and 12 genes (7 SNPs and 5 indels) in patient 2B. These non-synonymous SNPs occurred in genes with potential involvement in alterations of the cell envelope, virulence factors, transcriptional regulators, detoxification, stress response, and in lipid, amino acid, and iron metabolism. SNPs were predicted in all sequenced isolates from each patient and subsequent isolates were compared to the first isolate: 1S–1 for patient 1S and 2B–1 for patient 2B. Comparison of orthologous genes across the serial clinical isolates identified indels leading to deletion and frameshift events. Patient 1S and 2B had SNPs and indels in shared genes: porin, *rrl*, *mmpL*4 *and tetR*.

#### 3.5.1 Genomic rearrangement in the Porin locus in patient 1S and 2B:

**Figure 4.**
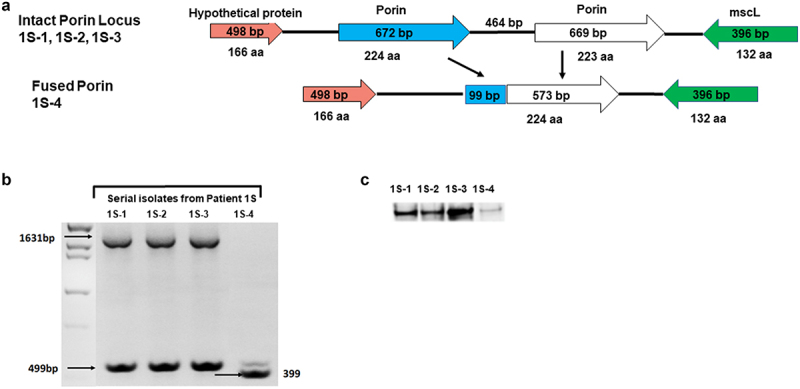
Recombination mutation in Porin locus in patient 1S: (a) Schematic view of the Porin locus in patient 1S isolates. The first three isolates from patient 1S (1S–1 to 1S–3), had two intact copies of the porin gene; the last isolate (1S–4) had an in frame fusion of 99 bp from the first copy of the porin gene to 573 bp from the second copy of the porin gene. (b) PCR amplification of the porin locus. Analysis of the PCR products by gel electrophoresis revealed a ~ 1.6kb and ~ 0.5 kb products for the intact porin isolates (1S–1, 1S–2, and 1S–3) and only a ~ 0.4kb fragment for the porin mutant isolate 1S–4. (c) Porin protein expression Protein extracts from 1S isolates were resolved by SDS-PAGE followed by western blot using a polyclonal anti-Porin (MspA) antibody. Isolate 1S–4 exhibited a significant reduction in porin protein expression.

There were significant genomic changes in the porin genes which are important for the diffusion of small and hydrophilic solutes across the outer membrane in mycobacterial isolates from both CF patients. For patient 1S, there was a deletion and in-frame merging of the tandem porin paralogs homologous to *M. abscesssus* ATCC 19,977^T^ genome MAB_1080 and MAB_1081 [10], resulting in a single copy of porin in the last isolate 1S–4 (Figure 4a). This deletion was confirmed by PCR using AShortF and Porin2B primers (Suppl. Table S3), in which ~ 1500 bp and ~ 500 bp PCR products were consistent with amplification of the region harbouring both porin tandem paralogs, which were present in 1S–1, 1S–2, and 1S–3. Loss of the ~ 1500 bp, suggestive of genomic changes in the porin region, was observed for isolate 1S–4 which, gave rise to a short product ~ 400 bp. (Figure 4b). Thus, we observed a recombination event in patient 1S where a 99 bp fragment from the 5’ end of the first copy of porin gene recombined with a 573 bp fragment of the second copy of the gene (Figure 4a, Porin gene mutation listed in Table 3).

**Figure 5.**
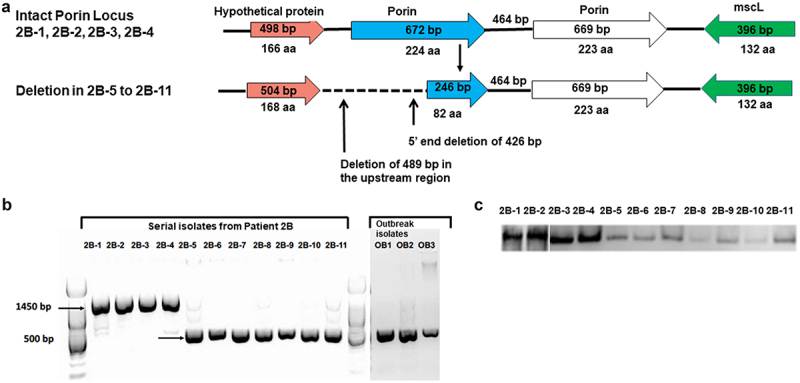
Partial deletion mutation in porin locus in patient 2B: (a) Schematic view of the Porin locus in patient 2B isolates. The first four isolates from patient 2B (2B–1 to 2B–4) had two intact copies of the porin gene; the last seven isolates (2B–5 to 2B–11) had a ~ 140bp deletion in the 5’end of the first porin paralog and a 489 bp deletion upstream. (b) PCR amplification of the porin locus. Analysis of the PCR products of patient 2B and Seattle outbreak isolates by gel electrophoresis showed a ~ 1.5kb for isolates with the intact locus and ~ 0.6 kb band for porin mutant strains. (c) Porin protein expression Protein extracts from 2B isolates were resolved by SDS-PAGE followed by western blot using a polyclonal anti-Porin (MspA) antibody. Isolate 2B–5 through 2B–11 exhibited a significant reduction in porin protein expression.

For patient 2B isolates, we observed a different mutation at the porin locus: a truncation event in the promoter region of the porin gene and a partial deletion of the first copy of porin (homologous to MAB_1080) gene resulting in a total deletion of ~ 900 bp in isolates 2B–5 through 2B–11 (Figure 5a). To assess the deletion event, a PCR using a primer located 652 bp upstream of the first porin gene 2B_0626_UP4F and Porin_int780RC (Suppl. Table S3) located in the intergenic region between the two copies the porin genes was developed. A PCR product of ~ 1449 bp was seen for the first four isolates, 2B–1 to 2B–4, and a shorter PCR fragment of ~ 534 bp was observed for isolates 2B–5, 2B–6, 2B–7, 2B–8, 2B–9, 2B–10, 2B–11, and outbreak isolates: OB1, OB2, OB3 (Figure 5b). The first 426 bp (142 amino acids) were deleted in the first paralog of porin along with a deletion of 489 bp in the upstream region, which were first noted in isolate 2B–5. Using the bacterial promoter search algorithm BPROM [38], a promoter region was identified ~ 479 bp upstream of the start codon of the first copy of porin and was included in the deletion identified in the upstream region. Three Seattle outbreak isolates, MAB_091912_2446 (OB1), MAB_091912_2455(OB2) and MAB_082312_2258 (OB3), for which Patient 2B was the index case [9] displayed the same porin deletion as found in isolate 2B–11, as shown by the short ~ 534 bp PCR product (Figure 5B, Porin gene listed in Table 4).

#### 3.5.3 SNPs in genes related to antibiotic resistancerrl gene 23S rRNA and 16S rRNA:

SNPs that occurred in isolates of both patient 1S and 2B included the 23S rRNA gene. A mutation A2058G in the 23S rRNA (*rrl*) gene was observed in the last isolate of patient 1S (1S–4, Table 1) and in isolates 2B–4 through the last isolate 2B–11 of patient 2B (Table 2) and correlated with acquired phenotypic clarithromycin resistance (Suppl. Table S2A and B) [32,33]. According to our records, anti-mycobacterial treatment (macrolides, amikacin, and ceftazidime) was initiated at a later stage in patient 1S’s disease course (isolate 1S–3) but early on (macrolides, linezolid and carbapenems) in patient 2B (isolate 2B–1).

**Table 1.** Variant analysis of non-synonymous SNPs across whole genome sequenced serial isolates from patient 1S compared to first isolate 1S–1.

Gene Name	Description	GO Biological process/Function	MAB Locus	Locus Tag First isolate (1S–1)	Amino acids	1S–1 smooth	1S–2 smooth	1S–3 smooth	1S–4 smooth, slightly rough	Mutation (AA change)	Occurrence of mutation (in years) from first positive culture
*rrl*	23S rRNA	n/a	MAB_r5052	MM1S1540310_0972	3112 bp	A	A	A	A → G	Clarithromycin Resistance (Base-position 2270 = 2058 in E coli rrl)	5.0
*fadD31*	Acyl-CoA synthetase	Metabolic process/transferase activity/fatty-acyl-CoA synthase activity/catalytic activity	MAB_1915	MM1S1540310_1347	600	G	G	G	G → T 10 G, 40T	W → C (398)	5.0
*mmpL4*	Putative membrane protein	Integral to membrane / Lipid transport	MAB_2037	MM1S1540310_1474	959	C	C	C	C → T	P → L (726)	5.0
*echs1*	Enoyl-CoA hydratase	Metabolic process/lyase activity/catalytic activity/enoyl-CoA hydratase activity	MAB_2058	MM1S1540310_1501	255	T	T	T	T → C	L → P (158)	5.0
*pdxp*	HAD (Haloacid dehalogenase) superfamily hydrolase	Metabolic process/pyridoxal phosphatase activity/catalytic activity/ phosphoglycolate phosphatase activity	MAB_2357	MM1S1540310_1793	334	C	C	C → T	T	R → o C (89)	4.0
*sis31*	Activator of Hsp90 ATPase 1 family protein	Response to stress	MAB_3864c	MM1S1540310_3492	161	C	C	C	C → A 6C, 9A	P → H (102)	5.0
Hypothetical protein	Putative drug-transport Integral membrane protein	Transmembrane transport	MAB_3908c	MM1S1540310_3501	438	A	A	A	A → C	M → R (117)	5.0
MFS transporter	Putative ABC transport system membrane domain protein	Transmembrane transport/transporter activity	MAB_0450c	MM1S1540310_4991*	421	C	C → A	A	A	R → L (4)	1.0
						G	G → A	A	A	A → V (2)	1.0
						C	C → A	A	A	V → F (308)	1.0

(* In isolate 1S–1, the putative ABC transport gene MM1S1540310_4991 is located on 2 contigs, there are 3 NSYNs between the 1S–1 and other 3 isolates).

**Table 2.** Variant analysis of non-synonymous SNPs across whole genome sequenced serial isolates from patient 2B compared to the first isolate 2B–1.

Gene Name	Description	GO Biological process/Function	Gene Locus	Locus Tag First isolate (2B–1)	Amino acids	2B–1 smooth	2B–4 smooth	2B–5 smoot h	2B–6 smoot h	2B–7 rough	2B–9 rough	2B–11 rough	Mutation (AA change)	Occurrence of mutation (in years) from first positive culture
	Hypothetical protein	Unclassified	MAB_0345	MM2B0626_0206	162	G	G	G	G	G	G	G→A 25 G, 40A	M→I (77)	7.5 y
*rrs*	16S rRNA	RNA	MAB_r5051	MM2B0626_1332	n/a	60A, 30 G	A → G	G	G	G	G	G	Aminoglycoside resistance (Base-position 1375 = 1408 E coli rrs)	0 y (first isolate)
*rrl*	23S rRNA	RNA	MAB_r5052	MM2B0626_1333	n/a	A	A → G	G	G	G	G	G	Clarithromycin Resistance (Base-position 2270 = 2058 E coli rrl)	2.5 y
*mmpL4*	Putative membrane protein mmpL4	Integral to membrane / Lipid transport	MAB_2303	MM2B0626_2095	997	C	C	C	C	C	C	C→T 25C, 60T	A→V (333)	7.5 y
*leuB1*	3-isopropylmalate dehydrogenase	Oxidation reduction/oxidoreductase activity/NAD binding/magnesium ion binding/3- isopropylmalate dehydrogenaseactivity/tartrate dehydrogenase activity	MAB_4419	MM2B0626_4288	350	G	G	G	G	G	G	G→A 25 G, 70A	G→D (177)	7.5 y
*ppnK*	Inorganic polyphosphate/ATP-NAD kinase	oxidation reduction/phosphorylation/NAD+ kinase activity/transferase activity/oxidoreductase activity	MAB_4739	MM2B0626_4600	386	T	T → G	G	G	G	G	G	V→G (16)	2.5 y
*hrpA*	ATP-dependent helicase	nucleic acid metabolic process/ATP-dependent helicase activity	MAB_0056c	MM2B0626_4817	1286	G	G	G	G	G → T	T	T	N→K (446)	5 y

The “A1408G” SNP in 16S rRNA gene is known to confer resistance to aminoglycosides [39], was observed only in all isolates of patient 2B (Table 2), which were phenotypically resistant to amikacin with MIC of >64 μg/ml (Suppl. Table S2B). Interestingly, mutational aminoglycoside resistance was detected from the first known isolate of patient 2B (2B–1) which displayed a mixed population of sequencing reads with 67% 1408A (wild-type) and 33% 1408 G (mutation) while 100% 1408 G reads were seen in later isolates throughout the last isolate 2B–11 (Table 2). Intriguingly, while mutational aminoglycoside resistance was detected in first isolate 2B–1, amikacin treatment was only documented to have started about 6 years later.

##### 3.5.4 SNPs and indels in the GPL locus genes mmpL4 and mps2 and transcriptional regulator tetR:

Mutations in a third gene, a putative membrane protein *mmpL*4 which is part of the GPL locus, were common between patient 1S and 2B with two MAB homologs (Tables 1 and 2). SNPs in *mmpL*4 occurred in different locations for each patient: A “C->T” SNP in *mmpL*4 (MAB_2037) occurred in the last isolate 1S–4 in patient 1S resulting in P726L amino acid change (Table 1). Similarly, a “C->T” SNP in *mmpL*4 (MAB_2303) in isolate 2B–11 at a different location was observed, resulting in a A333V amino acid change. However, mixed populations of wild-type and mutant were observed for *mmpL*4 gene (29% “C” and 71% “T”) in isolate 2B–11 (Table 2).

In patient 2B from isolates 2B–7 to 2B–11, we observed a frameshift mutation in non-ribosomal peptide synthetase *mps*2 (MAB_4098c) resulting in two ORFs (Table 4). This *mps2* mutations was only observed in strains from patient 2B and correlated with rough colony morphology of 2B-7 - 2B-11.

In addition, indel mutations in *tetR* occurred in the same MAB homolog (MAB_1881c) in both patient 1S and 2B isolates (Tables 3 and 4). A frameshift mutation was observed in *tetR* which is a transcriptional regulator was observed in isolates 1S–2, 1S–3, and 1S–4 (patient 1S, Table 3). Different mutations in *tet*R were observed in isolates from patient 2B: a frameshift mutation was detected in strain 2B–5, while a gene truncation occurred in isolates 2B–4, 2B–6, 2B–7, 2B–9, and 2B–11 isolates (Table 4).

**Table 3.** Comparison of syntenic orthologs of patient 1S isolates by whole genome alignments to the first isolate 1S–1: Identification of insertions, deletions, truncations, and frame shift mutations in orthologous genes.

Gene Name	Description	GO Biological process/Function	MAB Locus	Locus Tag First isolate (1S–1)/last Isolate (1S–4) Ortholog ORFs	Amino acids (in isolate)	Attribute	Process
*esxH*	ESAT-6-like protein esxH	Protein binding	MAB_2228c	MM1S1540310_1666 (1S–1)/ none in 1S–4	96 (1S–1)	Deletion in 1S–2, 1S–3, and 1S–4	Deletion
*ppe8*	PPE family protein	Unclassified	MAB_2230c	MM1S1540310_1669 (1S–1)/ none in 1S–4	166 (1S–1)	Deletion in 1S–2, 1S–3, and 1S–4	Deletion
*mspA*	Porin	Porin activity	MAB_1081	MM1S1540310_0607 (1S–1)/ none in 1S–4	214 (1S–1)	Deletion in 1S–4	Deletion
Hypothetical protein	LppS family protein	Transferase activity	MAB_4537c	MM1S1540310_4181 (1S–1)/MM1S1510930_4626 and MM1S1510930_4627 (1S–4)	333 (1S–1) 259 and 52 (1S–4)	2 ORFs in 1S–4	In-frame stop codon
*tetR*	Transcriptional regulator TetR family	DNA binding	MAB_1881c	MM1S1540310_1311 (1S–1)/MM1S1510930_1750 and MM1S1510930_1751 (1S–4)	210 (1S–1) 58 and 160 (1S–4)	2 ORFs in 1S–2, 1S–3, and 1S–4	Frameshift
*ppx3*	Exopolyphosphatase	Hydrolase activity/exopolyphosphatase activity	MAB_2061c	MM1S1540310_1503 (1S–1)/MM1S1510930_1947 and MM1S1510930_1948 (1S–4)	576 (1S–1) 418 and 119 (1S–4)	2 ORFs in 1S–2, 1S–3, and 1S–4	Frameshift
Iron-sulphur cluster-binding protein	Rieske (2Fe-2S) domain protein	Unclassified	MAB_0156c	MM1S1540310_4699 and MM1S1540310_4700 (1S–1)/MM1S1510930_5144 (1S–4)	120 and 408 (1S–1) 518 (1S–4)	2 ORFs in 1S–1	Frameshift

**Table 4.** Comparison of syntenic orthologs of genes of patient 2B isolates by whole genome alignments to the first isolate 2B–1: Identification of insertions, deletions, truncations, and frame shift mutations in orthologous genes.

Gene Name	Description	GO Biological process/Function	MAB Locus	Locus Tag First isolate (2B–1)/Last Isolate (2B–11) Ortholog ORFs	Amino acids (in isolate)	Attribute	Process
*esxS*	PE family protein	Protein binding	MAB_2229c	None in 2B–1/MM2B0107_1362 (2B–11)	98 (2B–11)	Additional ORF in isolate 2B–11	Insertion
*mspA*	porin	Porin activity	MAB_1080	MM2B0626_0880 (2B–1) MM2B0107_0216 (2B–11)	224 (2B–1) 82 (2B–11)	ORF truncated from isolates 2B–5 to 2B–11	Truncation
*tetR*	Transcriptional regulator, TetR family	DNA binding, Transcription, DNA- dependent	MAB_1881c	MM2B0626_1667 (2B–1)/MM2B0107_1006 (2B–11)	210 (2B–1) 154 (2B–11)	ORF truncated from isolates 2B–4, 2B–6, 2B–7, 2B–9, and 2B–11. 2B–5 has 2 ORFs 149 and 66 amino acids	Partial deletion, Frameshift
*mps2*	Non-ribosomal peptide synthetase	Biosynthetic process/phosphopantetheine binding/cofactor binding/acyl carrier activity/hydrolase activity, ligase activity	MAB_4098c	MM2B0626_3990 (2B–1)/MM2B0107_3328 and MM2B0107_3329 (2B–11)	2582 (2B–1) 267 and 2327 (2B–11)	2 ORFs in isolate 2B–7, 2B–9, and 2B–11	Frameshift
*sucB*	dihydrolipoyllysine- residue succinyltransferase	dihydrolipoyllysine-residue succinyltransferase activity	MAB_1945c	MM2B0626_1741 (2B–1)/MM2B0107_1078 and MM2B0107_1079 (2B–11)	572 (2B–1) 241 and 305 (2B–11)	2 ORFs in isolates 2B–4, 2B–5, 2B–6, 2B–9, and 2B–11	Stop codon mutation

### 3.6. Other genomic changes

Further, we observed deletions in the following genes considered as virulence factors in patient 1S from isolates 1S–2, 1S–3, and 1S–4: ESAT-6-like protein *esxH*, PPE family protein *ppe8* (Table 3). Non-synonymous SNPs in isolates from patient 1S included genes in metabolic processes: Acyl-CoA synthetase *fadD*31, Enoyl-CoA hydratase *echs1*, Haloacid dehalogenase superfamily hydrolase *pdxp*, activator of Hsp90 ATPase 1 family protein *sis31*, a putative drug transport and a putative ABC transport system (Patient 1S, Table 1). Most of the SNPS listed above arose in the last isolate 1S–4. Interestingly, SNP analysis revealed the presence of mixed populations including *fadD*31 20% “C” and 80% “T” at position 398 and *sis31* 40% “C” and 60% “A” at position 102. Of note, 3 NSYNs were detected in a putative ABC transport gene in isolate 1S–2 persisting throughout the last isolate 1S–4 (Patient 1S, Table 1).

Non-synonymous SNPs in isolates from patient 2B included the following genes related to metabolic processes: 3-isopropyl malate dehydrogenase *leuB1* in 2B–11, inorganic polyphosphate/ATP-NAD kinase *ppnK* from isolate 2B–4, and ATP-dependent helicase *hrpA* from isolate 2B–7. Mixed populations were observed for *leu*B1 (26% “G” and 74% “A”) in isolate 2B–11 (Patient 2B, Table 2). An indel resulting from a stop codon mutation was observed in dihydroliposyllysine-residue succinyltransferase *sucB* from isolates 2B–4 to 2B–11. Further, an insertion of a PE-family protein EsxS (*esxS)* was present in 2B–11 but absent in 2B–1 (Patient 2B, Table 4).

### 3.7. Evaluation of porin protein function

To investigate the impact of the deletion in the porin gene on protein expression, membrane fractions from lysates of these isolates were assayed by immunoblotting using an anti-Porin antibody [31]. Immunoblots showed a strong band of the porin protein detected in all clinical isolates from patient 1S: 1S–1, 1S–2, 1S–3, except for the last isolate 1S–4 which showed a significantly reduced expression level (Figure 4c). Similarly, patient 2B isolates with the altered porin locus showed markedly reduced expression of the protein from isolates 2B–5 to 2B–11 (Figure 5c) compared to that of earlier strains.

**Figure 6.**
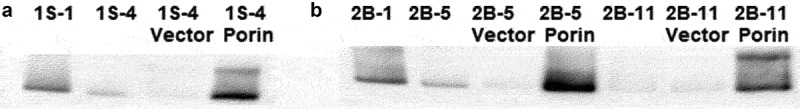
(a) Porin protein expression of porin complemented 1S–4 strain. Western blot with anti-Porin (MspA) antibody were performed on isolates 1S–1, 1S–4, and 1S–4 complemented with porin locus or vector control. (b) Porin protein expression of porin complemented 2B–5 and 2B-11strains Western blot with anti-Porin (MspA) antibody were performed on isolates 2B–1, 2B–5, 2B–11, and 2B–5 and 2B–11 complemented with the porin locus or vector control.

We sought to determine whether the observed genomic changes in the porin locus from patient 1S and 2B impacted porin function. We used^14^C-labelled glucose uptake as a measure of porin channel activity. For patient 1S, ^14^C glucose uptake in the last isolate (1S–4) was significantly reduced compared to the first isolate (1S–1) at 15 minutes (p-value <0.01), 20 minutes (p-value <0.04) and 25 minutes (p-value <0.0001) (Suppl. Figure S3A). Upon complementation of isolate 1S–4 with a Porin plasmid (1S–4 Porin), the ^14^C glucose uptake capacity was restored to 69.3–89.8% of wild type 1S–1 at time points 15 to 25 minutes, respectively (Suppl. Figure S3B). Glucose uptake in isolate 1S–4 complemented with vector alone remained reduced compared to 1S–1 at 15 and 25 minutes (p-value <0.0001). In addition, porin complementation of 1S–4 restored porin protein expression as evaluated by western blot (Figure 6a).

For patient 2B, ^14^Cglucose uptake in 2B–5 (first porin mutant) and in the last isolate, 2B–11, was significantly reduced at 5, 10, 15, 20 and 25 minutes compared to the first isolate, 2B–1 (p-value <0.0001) (Suppl. Figure S4A). Complementation of 2B–5 with the porin gene (2B–5 Porin), showed 50–65% recovery of wild type ^14^Cglucose uptake at 10, 15, 20 and 25 min (p-value <0.05) (Suppl. Figure S4B). Porin complementation of the last isolate, 2B–11, which displayed a very rough colony morphology, showed no change in the ^14^CGlucose uptake (Suppl. Figure S4C). Western blot analysis confirmed porin expression in porin complemented isolates 2B–5 and 2B–11 (Suppl. Figure S6B).

### 3.8. *Early and late clinical isolates of* M. massiliense *induce different inflammatory profiles* in vitro

**Figure 7.**
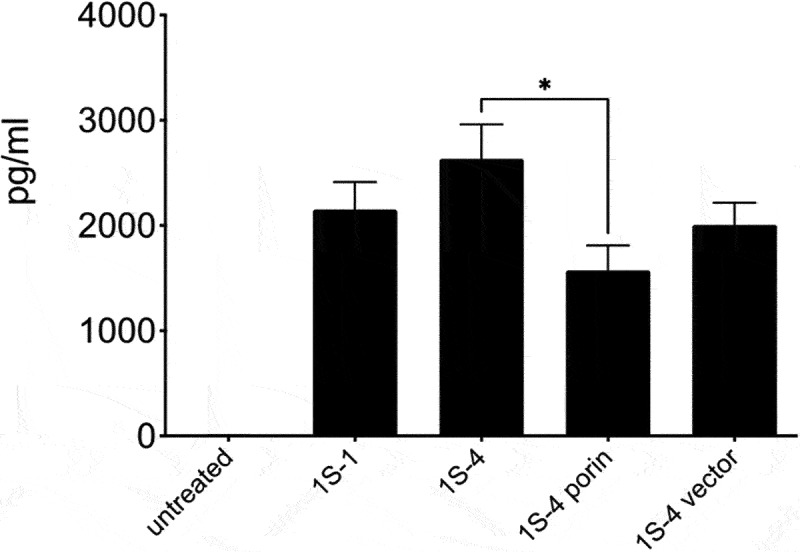
Cytokine response of THP-1 cells infected with Patient S1 strains and porin complemented strains: TNF-α levels (pg/ml) were determined on 24 h culture supernatants using a bead-based cytokine assay. Results are mean ± SEM from a total of 5 independent experiments. Complementation of 1S–4 with the porin containing plasmid (1S–4 porin) led to a reduction in TNF-α (* p-value 0.0160, FDR of 0.05).

**Figure 8.**
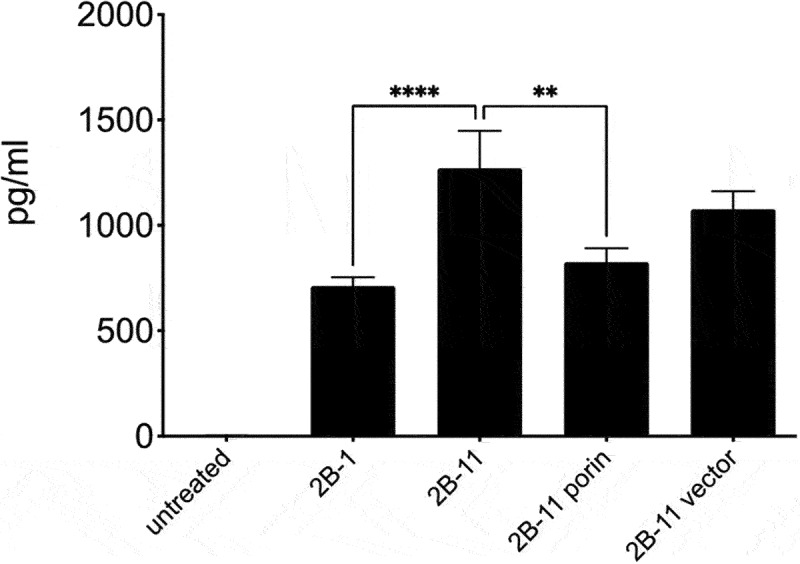
Cytokine response of THP-1 cells infected with Patient 2B strains and porin complemented strains: TNF-α levels (pg/ml) were determined on 24 h culture supernatants using a bead-based cytokine assay. Results are mean ± SEM from a total of 5 independent experiments. TNF-α levels were significantly higher in patient 2B last isolate 2B–11 compared to the first strain 2B–1 (**** p-value 0.0001, FDR of 0.05). Porin complementation of 2B–11 resulted in a reduction in TNF-α (** p-value 0.0040, FDR of 0.05).

We sought to investigate differences in bacterial-induced human cell inflammatory profiles *in vitro* among isolates collected at early or late stages of patients’ clinical courses. Differentiated human monocytic THP-1 cells were stimulated with first (1S–1 and 2B–1) and last isolate (1S–4 and 2B–11) at MOI of 5. The last isolate from patient 1S (1S–4) induced more TNF-α than the first isolate (1S–1), although the difference was not statistically significant. For patient 2B, the last isolate 2B–11 induced much more TNF-α than the first isolate 2B–1 (p-value 0.0001, FDR of 0.05). Furthermore, complementation of isolate 1S–4 with the porin containing plasmid (1S–4 porin) led to a reduction in TNF-α levels which were statistically significant (Figure 7) (p-value 0.0160, FDR of 0.05). Complementation of 2B–11 with the porin plasmid resulted in a reduction in TNF-α (p-value 0.0040, FDR of 0.05) to a level like the first isolate 2B–1 (Figure 8). IL-1 beta and IL-6 cytokine levels were not significantly different among the first and last isolates: 1S–1 and 1S–4; and 2B–1 and 2B–11 (data not shown).

## 4. Discussion

Whole genome sequencing, phenotypic assessment, and antibiotic susceptibility testing of the strains identified cumulative changes over the patient’s clinical courses (5–8 years) from stable condition to clinical decline and death. In our study, whole genome sequencing analysis of serial *M. massiliense* identified two intact porin genes homologous to *M. abscessus* ATCC 19977^T^ MAB_1080 and MAB_1081 Msp-type porins in early isolates with detectable level of expression and porin activity. *M. smegmatis* contains four porin genes (*msp*A-D) with *msp*A as the major porin [40]. There were two differing events in patients 1S and 2B in the porin locus (corresponding to MAB_1080, MAB_1081) genes. A fusion of the two copies of porin genes was observed in 1S–4 or the short indel observed in isolates 2B–5 through 2B–11 were also found in other publicly available genomes and have been previously described [41]. In our study, strains harbouring porin mutations (1S–4, 2B–5, 2B–6, 2B–7, 2B–8, 2B–9, 2B–10, and 2B–11) showed markedly reduced porin expression and impaired glucose intake. They also displayed slower *in vitro* growth rate consistent with their decreased influx of nutrients. The reduced growth rate and impaired glucose uptake observed in our naturally occurring porin mutant mycobacteria isolates from patients 1S and 2B support the published data on engineered porin deletions in *M. smegmatis* [15] and *M. chelonae* [28]. Deletion of three (out of four) porin genes *mspA*, *mspC* and *mspD* significantly increased survival of *M. smegmatis* in J774 macrophages [16]. Recently de Moura and colleagues [41] demonstrated that a synthetic allelic replacement of the two porin genes in *M. abscessu*s subsp. *massiliense* CIP108297 decreased the growth rate and reduced glucose uptake [41].

SCID mice were recently challenged with wild type, synthetic porin mutants and porin complemented synthetic porin mutant strains. The synthetic porin mutant produced significantly higher bacterial load in multiple organs compared to the wild type isolate or porin complemented mutant. Furthermore, lung histopathology revealed a more severe inflammation with early granuloma formation with the porin mutant strain [41]. Our study of mycobacterial isolates with naturally occurring porin and other mutations within patients provides information of the effect of bacterial changes in the patient clinical status. FEV_1_ measurements were used as indicators of clinical status of patients with cystic fibrosis. For patient 1S, the lowest FEV_1_ value of 1.58 L was recorded at the time of the porin deficient last isolate 1S–4 was collected. For patient 2B, a very mild decline to FEV_1_ 2.19 L occurred around the time when the first porin deficient strain 2B–5 was collected. This was followed by another decrease in FEV_1_ to 1.95 L that was associated with a transition from smooth to rough morphology by the time the rough isolate 2B–7 was co-isolated along smooth isolate 2B–6. Although patient 2B received a lung transplant 74 days- prior to death, there was no recovery in the FEV_1_ value and patient 2B’s health continued to deteriorate. Based on available clinical history with other lung pathogens occurring much earlier in the patient clinical course, we believe that the changes in pulmonary function observed in patient 1S (Supplementary Figure S1A) and in 2B patient (Supplementary Figure S1B) are related to the *in vivo* evolution of the mycobacterial pathogen during chronic infection.

In support of our findings of porin gene rearrangements, analysis of WGS raw data of mycobacterial isolates from 15 of 190 CF patients were found to vary in the number of porin genes encoded at the porin locus [41]. In addition, a recent longitudinal study of 30 *M. abscessus* from 11 CF patients found porin gene changes in three isolates from one patient [42]. Previous work by Bryant, *et* *al*. [9] established same patient’s isolates differed by 20 to 38 SNPs [43]. Serial isolates for patient 1S and 2B differed by less than 38 SNPs (within each patient) consistent with persistence of a distinct mycobacterial lineage within each individual. Clones shown to be transmissible often occur in patients that have been exposed to multiple rounds of antibiotic therapy, with high rates of antibiotic resistance mutations in 23S rRNA and 16S rRNA found in the *M. massiliense* cluster as previously described [43]. In the same vein, Patient 2B was the index case at the CF transplant centre in Seattle [6] and those isolates are part of a *M. massiliense* cluster of dominant circulating clones (DCC) which can have high rates of constitutive amikacin and/or macrolide resistance [43].

Constitutive macrolide resistance is thought to occur following extended antibiotic exposure [33] and is the predominant macrolide resistance mechanism in *M. massiliense*, as most strains lack a functional inducible macrolide resistance *erm*(41) gene. The A2058G clarithromycin resistance mutation in the 23S rRNA gene was observed in isolate 1S–4 (patient 1S) and from 2B–4 (patient 2B) after exposure to macrolides. In contrast, the aminoglycoside resistance A1408G SNP in the 16S rRNA gene appeared in 33% of sequencing reads in a mixed population of wild-type and mutant 2B–1 (first isolate of patient 2B), even though records available showed amikacin administration occurred much later, around the time when isolate 2B–8 was isolated. It is possible that patient 2B received amikacin prior to isolation of strain 2B–1 (but not recorded in the medical records) leading to a mixed population of wild type and mutant 16S rRNA gene isolates. In addition, the patient could have had an earlier isolate (susceptible to amikacin) obtained prior to isolation of 2B–1. Alternatively, the patient’s isolate may have acquired amikacin resistance through horizontal gene acquisition by environmental clones possibly within a hospital patient population, and thus resulting in clones that are more efficient at infection and or transmission [43]. Further, the complexity of mixed populations of a given gene such as 16S rRNA in the lung underlies the critical need for clinical microbiology to detect antibiotic resistant populations leading to an early therapeutic intervention before the emergence of a fully resistant population. This could be achieved by targeted DNA sequencing of a panel of antibiotic resistant genes or use of digital droplet PCR in the clinical setting [44].

It has also been demonstrated that small and hydrophilic antibiotics, especially β-lactam antibiotics use porins for entering the cell in *M. smegmatis* [12]. We observed a 4-fold increase in MIC value for imipenem (β-lactam antibiotic) in porin deficient 1S–4 compared to the wild type isolate 1S–1. Previously, de Moura *et* *al.* have reported a 2 to 4-fold increase in resistance to imipenem in the knockout mutant of the first porin gene from *M. massiliense* CIP108297 [41]. Complementation with a porin in 1S–4 isolate showed a mild, 2-fold reduction in MIC for imipenem, while 2B–5 complemented with porin showed an 8-fold reduction in tigecycline MIC which was like 2B–1. However, there was no reduction in drug MIC values when 2B–11 was complemented with porin. Similarly, porin function, measured by glucose uptake was not restored by complementation in isolate 2B–11. Lack of functional porin activity upon porin complementation of this strain might be attributed to the altered constitution of the mycobacterial envelope in the absence of GPL, which may interfere with the assembly and/or function of the porin in 2B–11.

Growth of serial clinical isolates on solid media revealed a switch from smooth to rough colony morphology in patient 2B isolates with isolate 2B–7 which persisted through the last isolate 2B–11. Rough morphology has been associated with cording phenotype, enhanced inflammatory properties, and the development of acute respiratory failure and worsening symptoms in a chronically infected patient [45–47]. The GPL locus gene *mps*2 has been previously implicated in *M. abscessus* isolates with rough morphology [48,49]. Isolate 2B–7 from patient 2B first displayed a single nucleotide deletion in the *mps*2 gene resulting in a frame shift mutation and early translation termination which persisted through 2B–11. Further, these rough isolates 2B–7 to 2B–11 had slower growth and showed loss of the lipidic molecule GPL (data not shown). In contrast, the last isolate from patient 1S (1S–4) showed no mutations in *mps*2, no loss of GPL (data not shown), but a subtle yet noticeable increase in roughness. Similarly, another common gene variation in the two patients 1S–4 and 2B–11 was observed in the *mmpL*4 gene, which has been implicated in synthesis and transport of glycopeptidolipids [49]. Interestingly, mutations in the *mmpL4* was not observed in earlier rough isolates of patient 2B such as 2B–7 and 2B–9.

The TetR family of transcriptional regulators act as chemical sensors to monitor the cellular environment of *M. abscessus* [50] and often act as repressors and interact with a specific DNA target to prevent transcription [51]. Different mutation events in the *tet*R gene (MAB_1881c) were observed in isolates from patient 1S and 2B. TetR transcriptional regulators are largely associated with resistance to antibiotic resistance and regulation of genes coding for small molecule exporters [51]. It has been reported that another homolog in the *tet*R gene family in *M. abscessus*, MAB_2299c was found to control the expression of two distinct MmpS-MmpL efflux pumps involved in the cross-resistance to clofazimine and bedaquiline [52,53].

It is well established that inflammation plays a critical role in CF lung pathology and disease progression. The CF airway contains increased concentrations of pro-inflammatory mediators such as TNF-α [54]. We hypothesize that porin changes in isolates 1S–4 and 2B–11 may have caused rearrangement of the cell envelope leading to exposure of pro-inflammatory molecules on the mycobacterial surface. Porin complementation of both 1S–4 and 2B–11 isolates lead to a reduction of TNF-α levels. Increased levels of TNF-α and other proinflammatory cytokines induced by porin deleted 1S–4 and 2B–11 may have contributed to higher morbidity and eventually mortality of these patients.

In summary, this study suggests that porin deletions contribute to host adaptation of *M. abscessus* as part of its evolution within the host. Genes gained and lost in the genome may help mycobacteria adapt and survive in a variety of unfavourable environments [55]. The emergence of porin deletions in mycobacterial isolates, may serve as predictor of a potential decline in patient clinical status. Reduced porin activity promotes survival and persistence for mycobacteria based on a change in nutrient source from sugars to cholesterol [56] upon limited nutrient transport across the membrane [12,15] and decreased susceptibility to nitric oxide [16] and increases antibiotic resistance.

Finally, we hypothesize that specific mutations accumulated and maintained over time in *M. massiliense*, including mutations shared among transmissible strains, may collectively lead to more virulent, better adapted lineages which can be potentially relevant in the context of cystic fibrosis and perhaps other susceptible hosts.

## Supplementary Material

Supplemental Material

## Data Availability

The authors confirm that the data supporting the findings of this study are available within the article, its supplementary materials and NCBI https://www.ncbi.nlm.nih.gov/nuccore/ under accession numbers AKUL00000000, AKUK00000000, AKUJ00000000, AKUI00000000, AKUM00000000, AKUO00000000, JARETB000000000, AKUW00000000, AKUV00000000¸ AKUU00000000, AKUN00000000, JAOW00000000, AYTF00000000, JAOV00000000, AYTA00000000.

## Abbreviations

ADC: albumin, dextrose and catalase, BAL: bronchoalveolar lavage, CF: cystic fibrosis, CFU: colony forming units, FEV_1_: serial forced expiratory volume in 1 second, GPL: glycopeptidolipid, ICU: intensive care unit, MOI: multiplicity of infection, MTB: *Mycobacterium tuberculosis*, SNP: Single nucleotide polymorphisms, ORF: Open reading frame, RGM: rapidly growing mycobacteria, NTM: Nontuberculous mycobacteria.
