# Supplementary material for: Evolution of Mycobacterium abscessus in the human lung: Cumulative mutations and genomic rearrangement of porin genes in patient isolates
Source: Virulence. 2023 Jun 4;14(1):2215602. doi: 10.1080/21505594.2023.2215602 (PMC10243398; doi:10.1080/21505594.2023.2215602)
Supplement: Supplemental Material [file KVIR_A_2215602_SM8081.zip › Supplementary_table1_03_09_2023.docx]

Supplementary Table S1. Identification of isolates used in this study

| **Date of isolate acquisition** | **Whole genome**  **sequenced** | **NCBI genome ID** | **Designated isolate name** | **NCBI accession number** |
| --- | --- | --- | --- | --- |
| **Patient 1S** | | | | |
| **3/2004** | Yes | 1S-154-0310 | 1S-1 | AKUL00000000 |
| **9/2004** | Yes | 1S-153-0915 | 1S-2 | AKUK00000000 |
| **9/2007** | Yes | 1S-152-0914 | 1S-3 | AKUJ00000000 |
| **9/2008** | Yes | 1S-151-0930 | 1S-4 | AKUI00000000 |
| **Patient 2B** | | | | |
| **6/2000** | Yes | 2B-0626 | 2B-1 | AKUM00000000 |
| **1/2002** | No | N/A | 2B-2 | N/A |
| **7/2002** | No | N/A | 2B-3 | N/A |
| **12/2002** | Yes | 2B-1231 | 2B-4 | AKUO00000000 |
| **3/2004** | Yes | MAB_030804_1651 | 2B-5 | JARETB000000000 |
| ***9/2005** | Yes | 2B-0912-S | 2B-6 | AKUW00000000 |
| ***9/2005** | Yes | 2B-0912-R | 2B-7 | AKUV00000000 |
| **2/2007** | No | N/A | 2B-8 | N/A |
| **3/2007** | Yes | 2B-0307 | 2B-9 | AKUU00000000 |
| **12/2007** | No | N/A | 2B-10 | N/A |
| **1/2008** | Yes | 2B-0107 | 2B-11 | AKUN00000000 |
| **Seattle outbreak** | | | | |
| **2008** | Yes | MAB_082312_2272 | OB-1 | JAOW00000000 |
| **2008** | Yes | MAB_091912_2446 | OB-2 | AYTF00000000 |
| **2008** | Yes | MAB_091912_2455 | OB-3 | JAOV00000000 |
| **2008** | Yes | MAB_082312_2258 | OB-4 | AYTA00000000 |

* These two isolates were derived from a respiratory sample obtained on the same day; 2B-0912-S, smooth colony morphology; 2B-0912-R, rough colony morphology.

N/A Not applicable. Not sequenced.
