# Supplementary material for: Evolution of Mycobacterium abscessus in the human lung: Cumulative mutations and genomic rearrangement of porin genes in patient isolates
Source: Virulence. 2023 Jun 4;14(1):2215602. doi: 10.1080/21505594.2023.2215602 (PMC10243398; doi:10.1080/21505594.2023.2215602)
Supplement: Supplemental Material [file KVIR_A_2215602_SM8081.zip › Supplementary_tableS2A_11_18_2022.docx]

Supplementary Table S2A: Antibiotic susceptibility using Thermofisher RAPMYCO on patient 1S Isolates

| **Drug** | **1S-1 (smooth)** | **1S-4 (smooth, slightly rough)** | **1S-4 porin (smooth slightly rough)** |
| --- | --- | --- | --- |
| **Amikacin** | 4 (S) | 4 (S) | 4 (S) |
| **Cefoxitin** | 32 (I) | 32 (I) | 32 (I) |
| **Ciprofloxacin** | 4 (R) | >4 (R) | >4 (R) |
| **Clarithromycin** | <0.06 3 days/0.5 (S)  14days | >16 (R) 3 days | >16 (R) 3 days |
| **Doxycycline** | 8 (R ) | 16 (R) | >16 (R) |
| **Imipenem** | 4 (S) | 16 (I) | 8 (I) |
| **Linezolid** | 8 (S) | 16 (I) | 16 (I) |
| **Moxifloxacin** | 8 (R) | 8 (R) | >8 (R) |
| **Tigecycline** | 0.5 | 0.25 | 0.25 |
| **Tobramycin** | 8 (R) | 4 (I) | 16 (R) |
| **Trimethoprim- sulfamethoxazole** | 8/152 (R) | 8/152 (R) | 8/152 (R) |
