# Supplementary material for: Evolution of Mycobacterium abscessus in the human lung: Cumulative mutations and genomic rearrangement of porin genes in patient isolates
Source: Virulence. 2023 Jun 4;14(1):2215602. doi: 10.1080/21505594.2023.2215602 (PMC10243398; doi:10.1080/21505594.2023.2215602)
Supplement: Supplemental Material [file KVIR_A_2215602_SM8081.zip › Supplementary_tableS2B_11_18_2022.docx]

Supplementary Table S2B: Antibiotic susceptibility using Thermofisher RAPMYCO on patient 2B Isolates

| **Drug** | **2B-1 (smooth)** | **2B-5 (smooth)** | **2B-5 porin (smooth)** | **2B-11 (very rough)** | **2B-11 porin (very rough)** |
| --- | --- | --- | --- | --- | --- |
| **Amikacin** | >64 (R) | >64 (R) | >64 (R) | >64 (R) | >64 (R) |
| **Cefoxitin** | 64 (I) | >128 (R) | >128 ( R) | >128 (R) | >128 ( R) |
| **Ciprofloxacin** | >4 (R) | >4 (R) | >4 (R) | >4 (R) | >4 (R) |
| **Clarithromycin** | 0.25(S) day 3/ 1 (S) day 14 | >16 (R ) day 3 | >16 (R) day 3 | >16 (R) day 3 | >16 (R) day 3 |
| **Doxycycline** | >16 (R) | >16 (R) | 16 (R) | >16 (R) | >16 (R) |
| **Imipenem** | 64 (R ) | >64 (R) | >64 (R) | >64 (R) | >64 (R) |
| **Linezolid** | 32 (R) | >32 (R) | >32 ( R) | >32 (R) | >32 ( R) |
| **Moxifloxacin** | >8 (R) | >8 (R) | >8 (R) | >8 (R) | 8 ( R) |
| **Tigecycline** | 0.5 | 2 | 0.25 | 0.12 | 0.5 |
| **Tobramycin** | 16 (R) | >16 (R) | >16 (R) | >16 (R) | >16 (R) |
| **Trimethoprim- sulfamethoxazole** | >8/152 (R) | >8/152 (R) | >8/152 (R) | 2/38 (S) | 2/38 (S) |
