# Supplementary material for: Evolution of Mycobacterium abscessus in the human lung: Cumulative mutations and genomic rearrangement of porin genes in patient isolates
Source: Virulence. 2023 Jun 4;14(1):2215602. doi: 10.1080/21505594.2023.2215602 (PMC10243398; doi:10.1080/21505594.2023.2215602)
Supplement: Supplemental Material [file KVIR_A_2215602_SM8081.zip › Supplementary_tableS3_11_18_2022.docx]

Supplementary Table S3: Primers for porin locus, 23S rRNA and 16S rRNA

| **Primers** | **Sequence** | **Gene** | **Product size (bp)** |
| --- | --- | --- | --- |
| AShortF | 5’- CGGTGTGCTGGCGGTCGGCGCTC-3’ | *porin* PCR and | ~1500 bp and ~500 |
|  |  | sequencing for patient 1S isolates | bp (intact porin).  ~400 bp (gene |
| Porin2B | 5’- CACCCTTCGGACCGGCG-3’ |  |  |
|  |  |  | fusion mutation) |
| 2B_0626_UP4F | 5’-TCGCGCGGTGTCGCGGGCATTTCGG-3’ | *porin* PCR and | ~1450 bp (intact |
|  |  | sequencing for patient 2B isolates and 4 outbreak isolates | porin). ~530 bp (partial gene deletion) |
| Porin_int780RC | 5’-CCCGACGTTGCCCCAAGA-3’ |  |  |
| PorinF_HindIII | 5’-CACCACAAGCTTAGAATGAAG  CTGTTGAGC-3’ | Porin locus amplification | ~1800 bp |
| Porin2R_HindIII | 5’-CACCCAAGCTTGAGGCAGA  GCTGCTCG-3’ |  |  |
| 23S_18 Forward | 5’-AGTCGGGACCTAAGGCGAG-3’ | 23S rRNA PCR | 1,525 bp |
| 23S_21 Reverse | 5’-TTCCCGCTTAGATGCTTTCAG-3’ |  |  |
| 23SrRNAF_207  Forward | 5’-AGCGAAATTCCTTGTCGGGT-3’ | 23S rRNA sequencing | 207 bp |
| 23SrRNAR_207  Reverse | 5’-CTGCTTCACAGTCTCCCACC-3’ |  |  |
| rrs1-F | 5′-ATGACGTCAAGTCATCATGCC-3′) | 16S rRNA PCR and sequencing | 341 bp |
| rrs1-R | 5′-AGGTGATCCAGCCGCACCTTC-3′) |  |  |
