# Supplementary figures and images for: Evolution of Mycobacterium abscessus in the human lung: Cumulative mutations and genomic rearrangement of porin genes in patient isolates
Source: Virulence. 2023 Jun 4;14(1):2215602. doi: 10.1080/21505594.2023.2215602 (PMC10243398; doi:10.1080/21505594.2023.2215602)

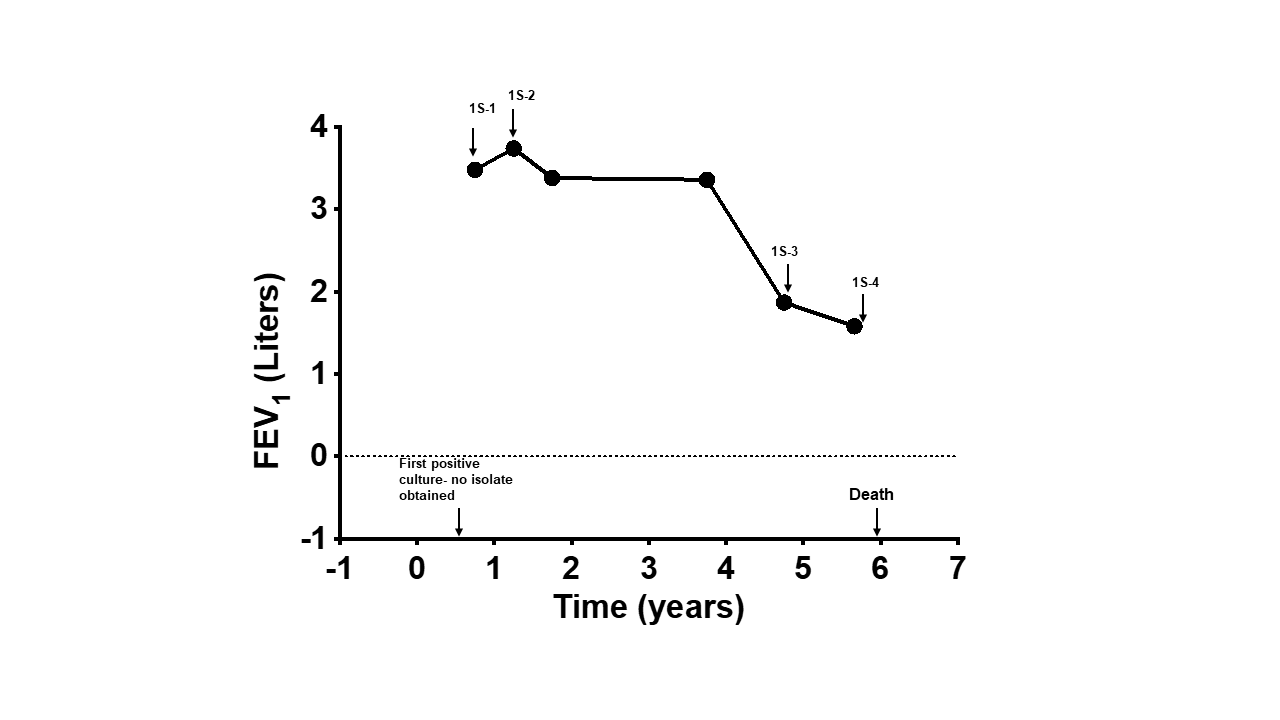

Supplement: Supplemental Material [file KVIR_A_2215602_SM8081.zip › Supplementary_Figure_1A_03-09-2023.tif]

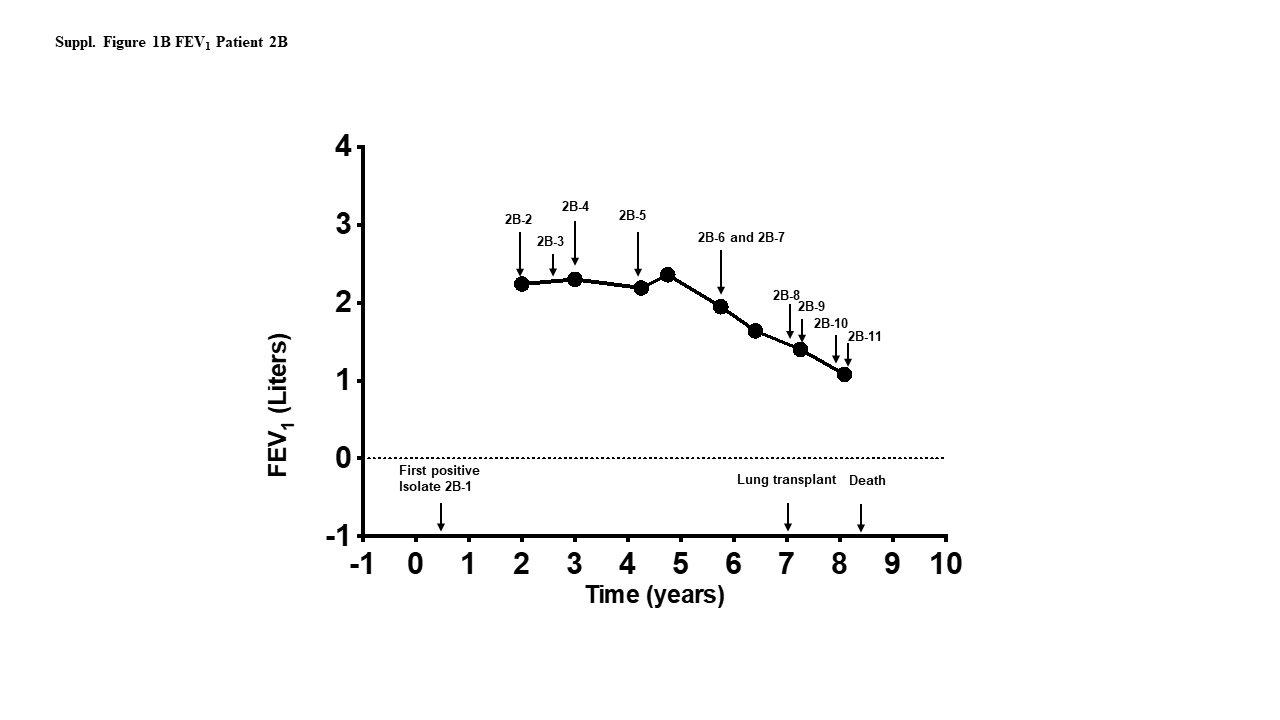

Supplement: Supplemental Material [file KVIR_A_2215602_SM8081.zip › Supplementary_Figure_1B_03-09-2023.tif]

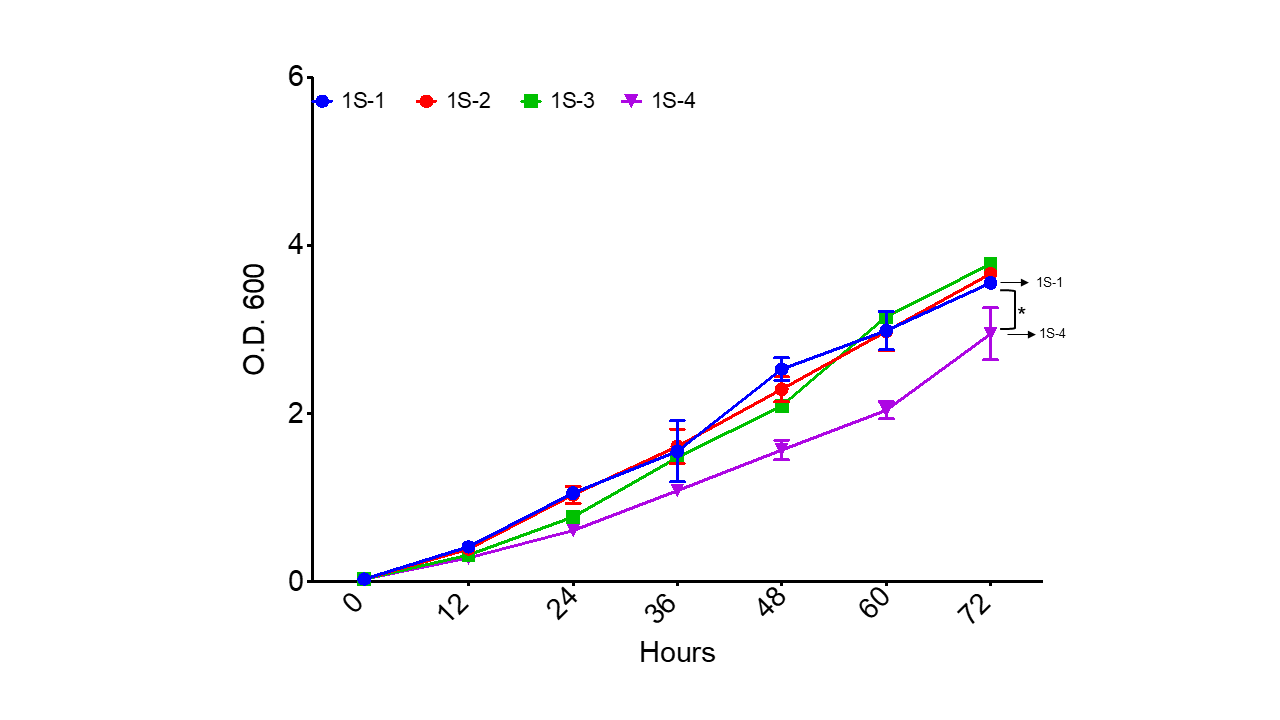

Supplement: Supplemental Material [file KVIR_A_2215602_SM8081.zip › Supplementary_Figure_2A_03-09-2023.tif]

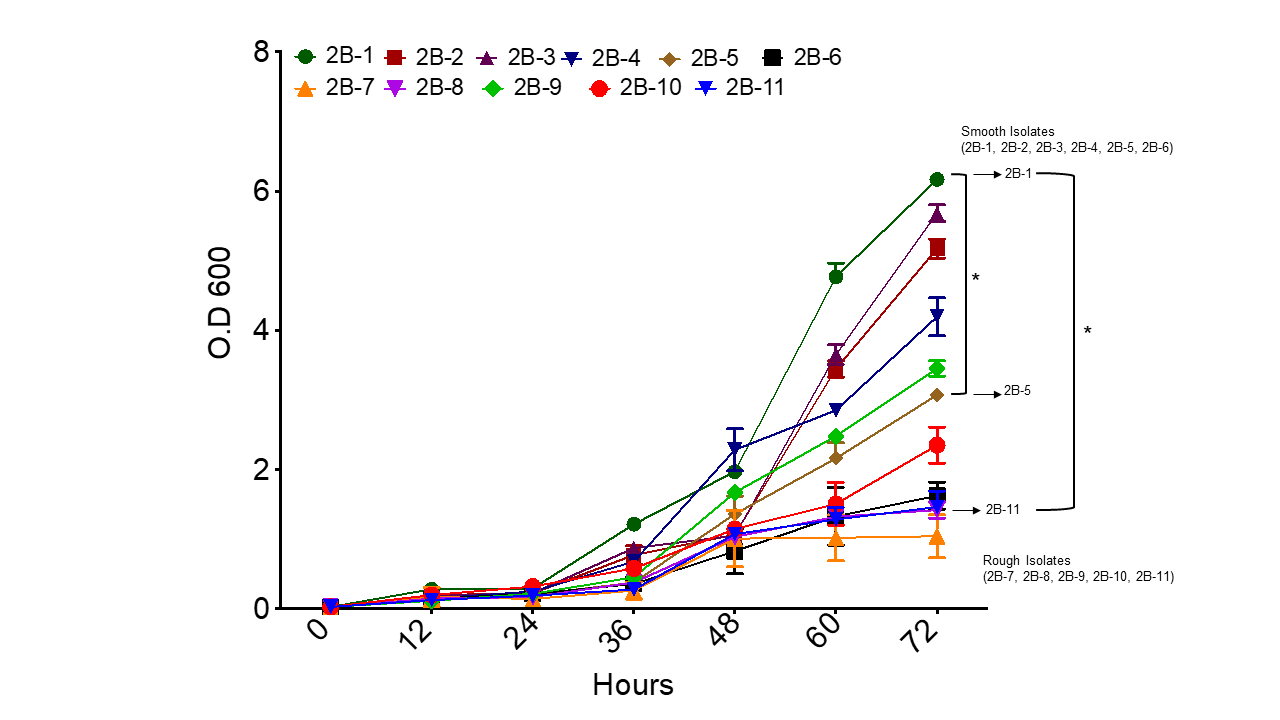

Supplement: Supplemental Material [file KVIR_A_2215602_SM8081.zip › Supplementary_Figure_2B_03-09-2023.tif]

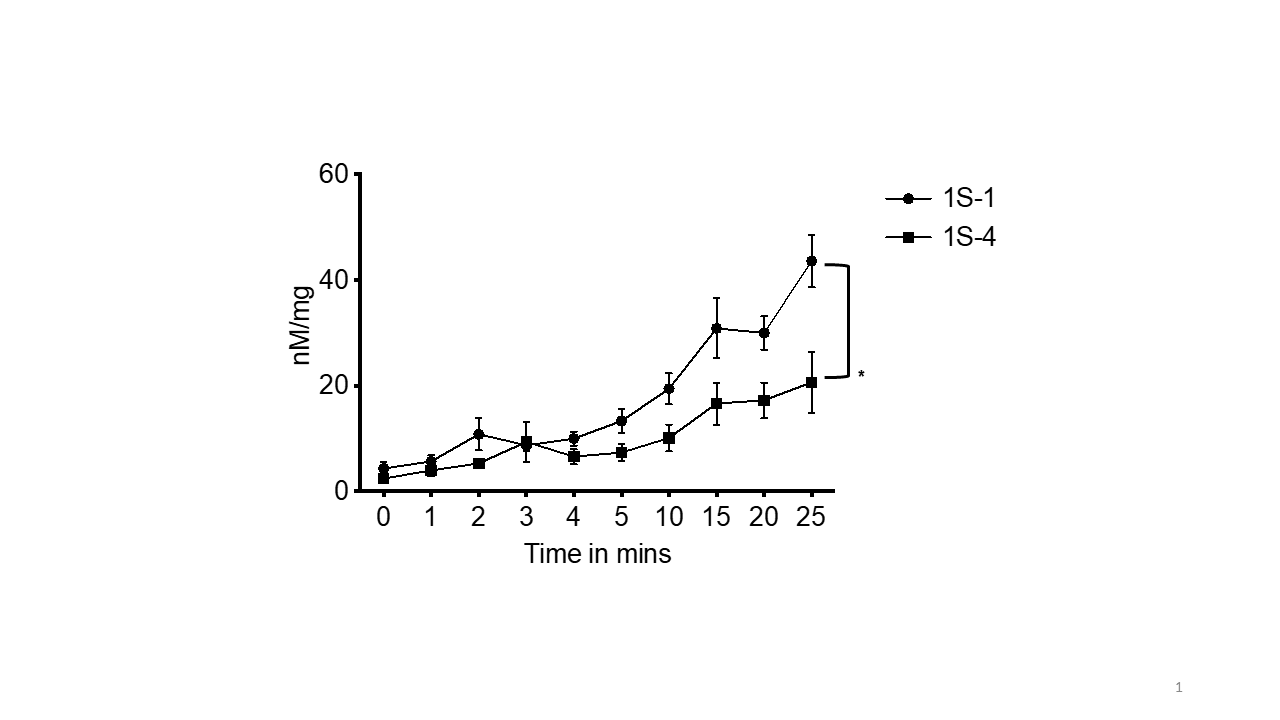

Supplement: Supplemental Material [file KVIR_A_2215602_SM8081.zip › Supplementary_Figure_3A_03-09-2023.tif]

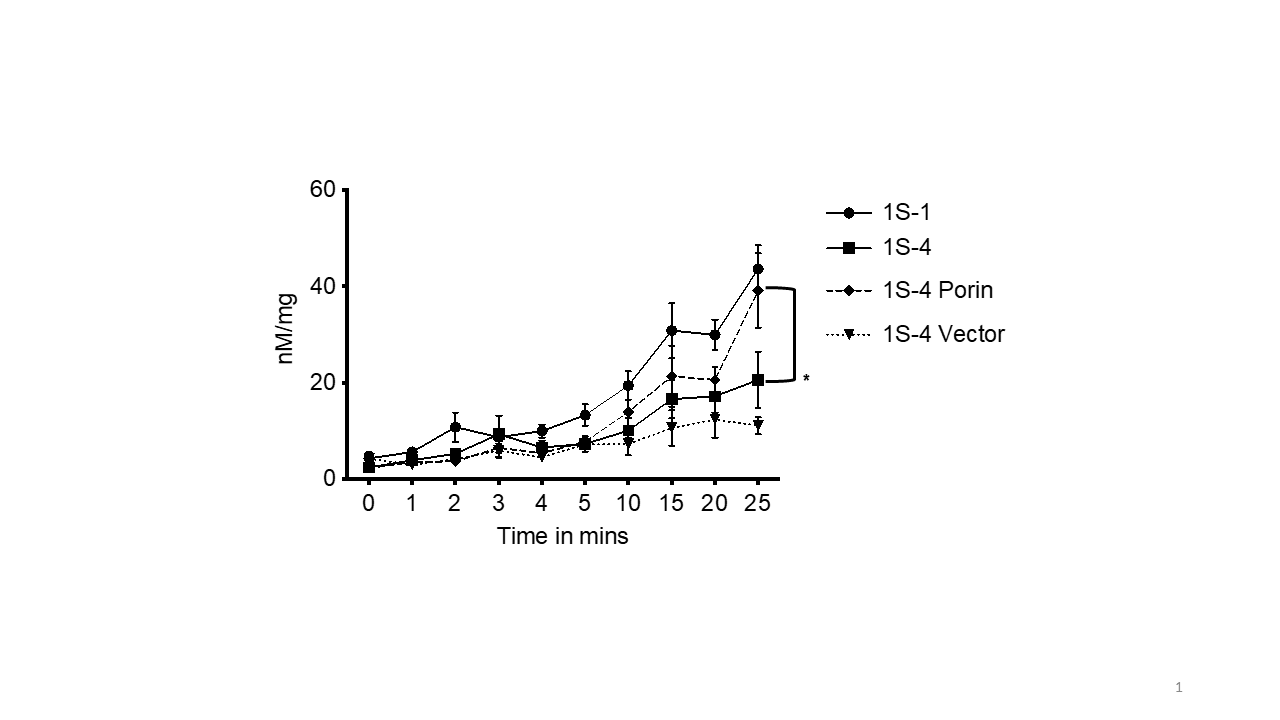

Supplement: Supplemental Material [file KVIR_A_2215602_SM8081.zip › Supplementary_Figure_3B_03-09-2023.tif]

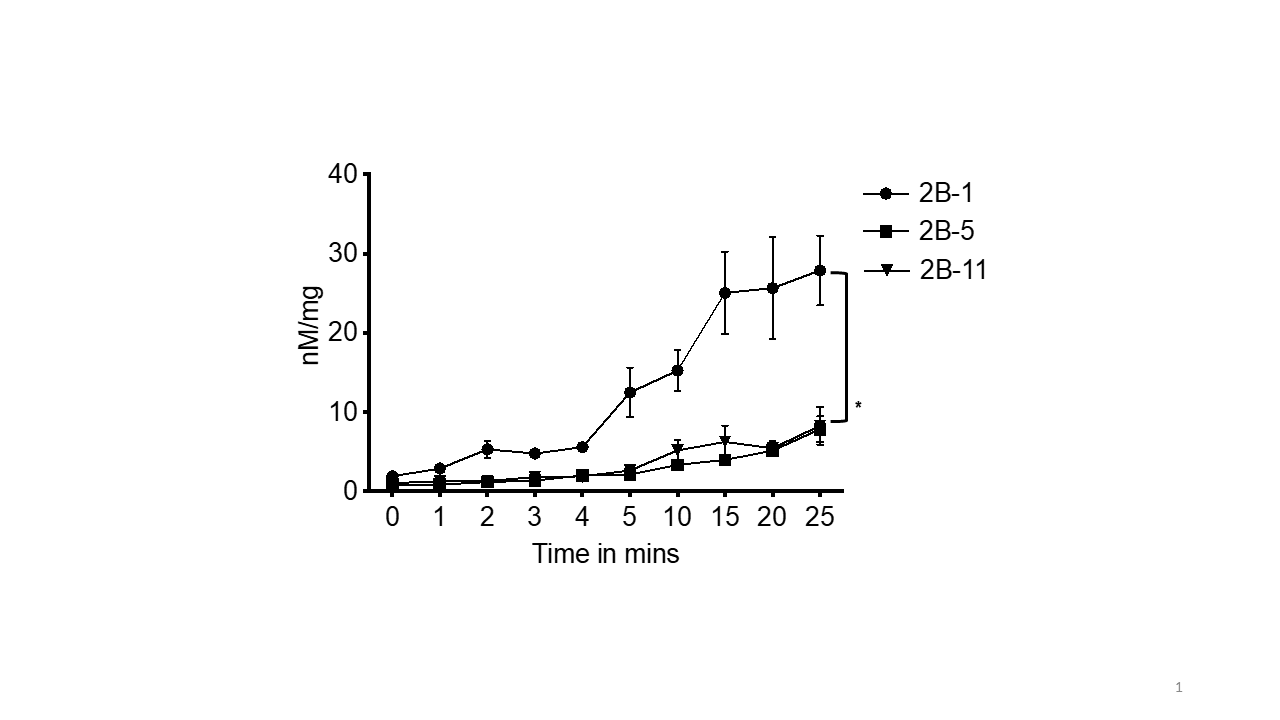

Supplement: Supplemental Material [file KVIR_A_2215602_SM8081.zip › Supplementary_Figure_4A_03-09-2023.tif]

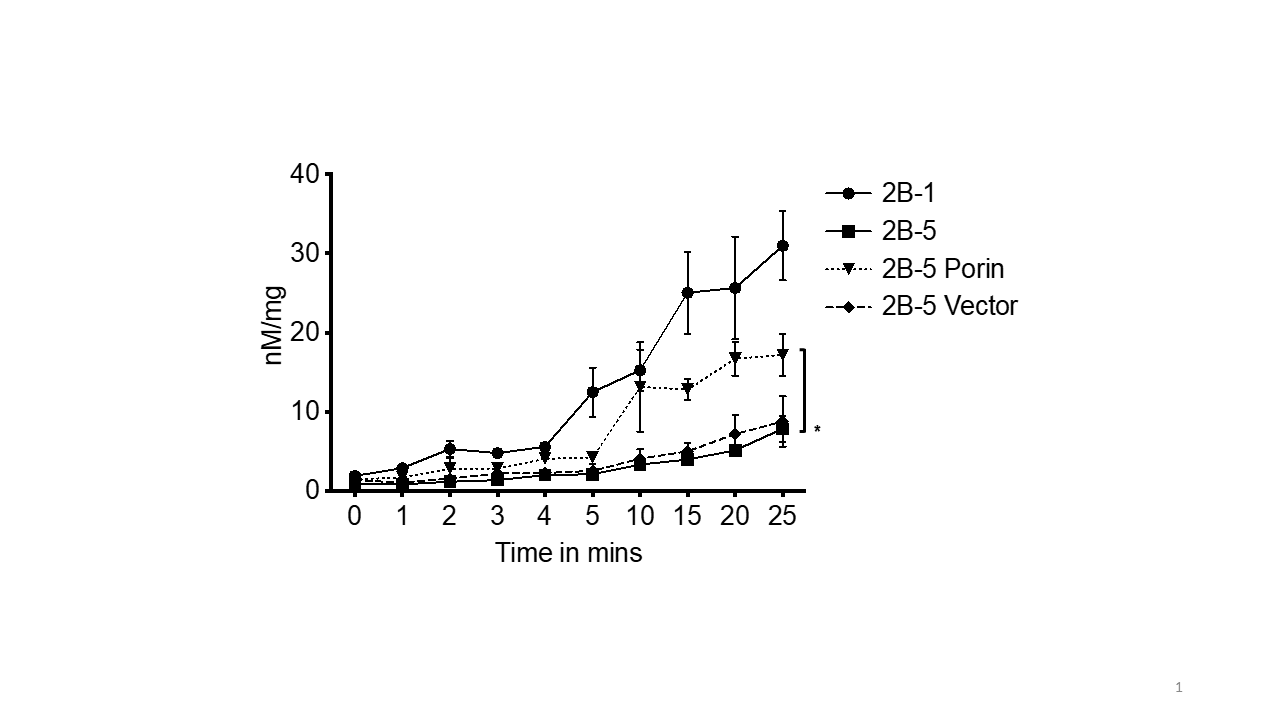

Supplement: Supplemental Material [file KVIR_A_2215602_SM8081.zip › Supplementary_Figure_4B_03-09-2023.tif]

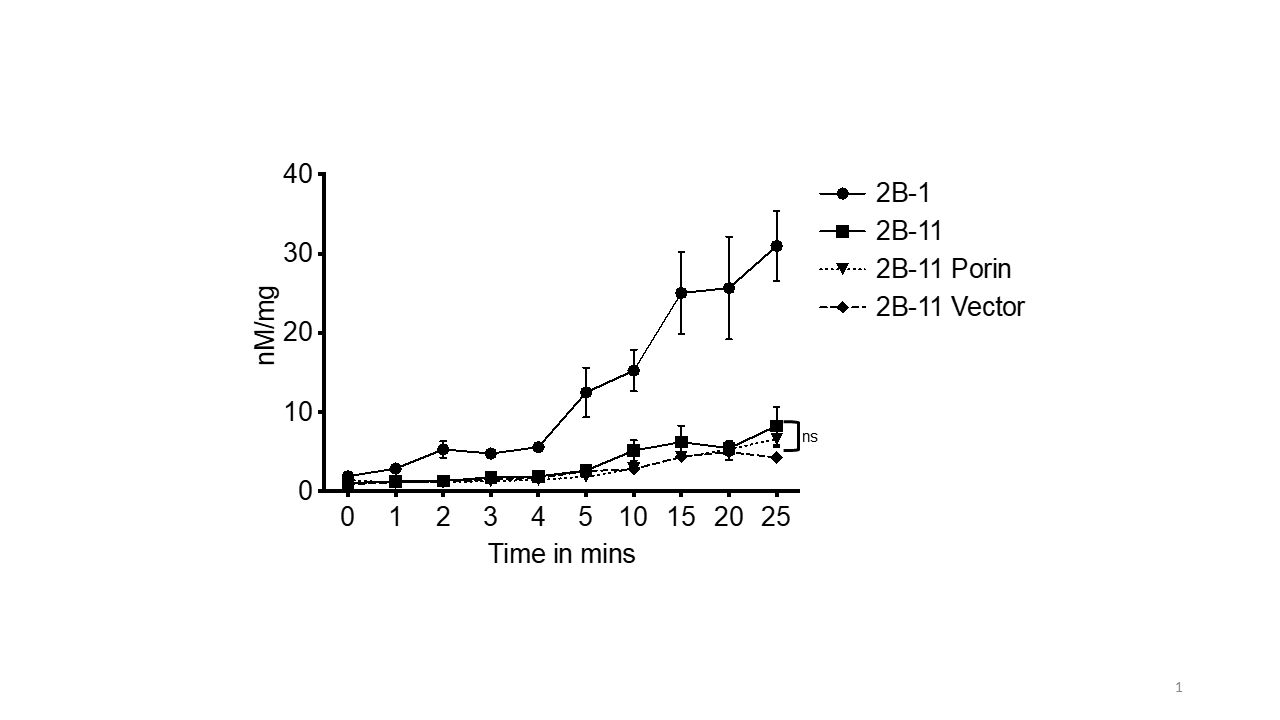

Supplement: Supplemental Material [file KVIR_A_2215602_SM8081.zip › Supplementary_Figure_4C_03-09-2023.tif]
